# MicroRNA-449a Inhibits Triple Negative Breast Cancer by Disturbing DNA Repair and Chromatid Separation

**DOI:** 10.3390/ijms23095131

**Published:** 2022-05-04

**Authors:** Beate Vajen, Rahul Bhowmick, Luisa Greiwe, Vera Schäffer, Marlies Eilers, Thea Reinkens, Amelie Stalke, Gunnar Schmidt, Jan Fiedler, Thomas Thum, David S. DeLuca, Ian D. Hickson, Brigitte Schlegelberger, Thomas Illig, Britta Skawran

**Affiliations:** 1 Department of Human Genetics, Hannover Medical School, Carl-Neuberg-Str. 1, 30629 Hannover, Germany; luisa.greiwe@gmail.com (L.G.); schaeffer.vera@mh-hannover.de (V.S.); eilers.marlies@mh-hannover.de (M.E.); reinkens.thea@mh-hannover.de (T.R.); stalke.amelie@mh-hannover.de (A.S.); schmidt.gunnar@mh-hannover.de (G.S.); schlegelberger.brigitte@mh-hannover.de (B.S.); illig.thomas@mh-hannover.de (T.I.); skawran.britta@mh-hannover.de (B.S.); 2 Center for Chromosome Stability, Department of Cellular and Molecular Medicine, University of Copenhagen, Blegdamsvej 3B, DK-2200 Copenhagen, Denmark; rahul@sund.ku.dk (R.B.); iandh@sund.ku.dk (I.D.H.); 3 Institute of Molecular and Translational Therapeutic Strategies (IMTTS), Hannover Medical School, Carl-Neuberg-Str. 1, 30629 Hannover, Germany; fiedler.jan@mh-hannover.de (J.F.); thum.thomas@mh-hannover.de (T.T.); 4 Fraunhofer Institute for Toxicology and Experimental Medicine (ITEM), Nikolai-Fuchs Str. 1, 30625 Hannover, Germany; 5 REBIRTH Center for Translational Regenerative Medicine, Hannover Medical School, Carl-Neuberg-Str. 1, 30629 Hannover, Germany; 6 German Center for Lung Research (BREATH), Hannover Medical School, Carl-Neuberg-Str. 1, 30629 Hannover, Germany; deluca.david@mh-hannover.de

**Keywords:** microRNA449a, triple negative breast cancer, DNA repair, chromosomal instability, chromatid separation

## Abstract

Chromosomal instability (CIN) can be a driver of tumorigenesis but is also a promising therapeutic target for cancer associated with poor prognosis such as triple negative breast cancer (TNBC). The treatment of TNBC cells with defects in DNA repair genes with poly(ADP-ribose) polymerase inhibitor (PARPi) massively increases CIN, resulting in apoptosis. Here, we identified a previously unknown role of microRNA-449a in CIN. The transfection of TNBC cell lines HCC38, HCC1937 and HCC1395 with microRNA-449a mimics led to induced apoptosis, reduced cell proliferation, and reduced expression of genes in homology directed repair (HDR) in microarray analyses. *EME1* was identified as a new target gene by immunoprecipitation and luciferase assays. The reduced expression of *EME1* led to an increased frequency of ultrafine bridges, 53BP1 foci, and micronuclei. The induced expression of microRNA-449a elevated CIN beyond tolerable levels and induced apoptosis in TNBC cell lines by two different mechanisms: (I) promoting chromatid mis-segregation by targeting endonuclease *EME1* and (II) inhibiting HDR by downregulating key players of the HDR network such as *E2F3*, *BIRC5*, *BRCA2* and *RAD51*. The ectopic expression of microRNA-449a enhanced the toxic effect of PARPi in cells with pathogenic germline *BRCA1* variants. The newly identified role makes microRNA-449a an interesting therapeutic target for TNBC.

## 1. Introduction

Breast cancer is the second leading cause of cancer-related death, and the most common malignancy in women worldwide. Among the different breast cancer subtypes, triple-negative breast cancer (TNBC), defined by the absence of estrogen and progesterone receptor and HER2 expression, accounts for 15–20% of all cases [1]. TNBC patients have relatively low survival rates as they often relapse within 1–3 years after diagnosis. 15–20% of TNBC patients harbor germline mutations in *BRCA1* and *BRCA2*, which makes them sensitive towards poly (ADP-ribose) polymerase (PARP) inhibitors. Breast cancer cells with defects in the homologous DNA repair machinery have to rely on PARP for DNA repair. However, for the majority of women with TNBC treatment options are limited [2]. Therefore, the identification of molecular targets and the rapid development of new therapeutic agents are critical for these patients.

In addition to BRCA1 and BRCA2, important players within the homology-directed repair (HDR) network are often affected in TNBC patients [3]. Survivin (coded by *BIRC5*, baculoviral IAP repeat-containing protein 5) is highly expressed in breast cancer cells. A recent study has shown that Survivin contributes to DNA repair by affecting the expression of various genes in the HDR pathway such as *BRCA1*, *BRCA2*, the recombinase *RAD51* as well as the endonuclease complex *MUS81/EME1* (essential meiotic endonuclease 1) [4]. This complex minimizes mis-segregation at difficult-to-replicate regions of the human genome [5]. The stabilization of EME1 is furthermore associated with resistance against monoclonal antibody therapies: Cetuximab initiates pathways that result in the stabilization of EME1, resulting in enhanced DNA repair and the reduction of the effectiveness of DNA-damaging therapies [6]. 

MicroRNA (miRNA) replacement is a relatively new treatment concept for various types of cancer. These small, non-coding RNAs post-transcriptionally regulate gene expression by binding to complementary sites at the 3′-untranslated regions (3′UTRs) of specific target mRNAs, which then initiates mRNA degradation [7]. The global downregulation of miRNAs in cancer, as demonstrated for miR-449a in TNBC, makes them an interesting therapeutic option [8]. We have previously reported that increased acetylation of the promoter of the host gene of miR-449 by treatment with histone deacetylase (HDAC) inhibitors led to the induced expression of miR-449 followed by apoptosis and the inhibition of migration in liver cancer. This effect was mediated by the downregulation of *c-Met* and *SOX4* [9,10]. Here, we present new targets of miR-449a, which support the proposed role in combating tumor progression in TNBC. We demonstrate the ability of miR-449a to increase chromosomal instability by promoting the mis-segregation of chromatids by targeting *EME1* and inhibiting HDR of DNA-double strand breaks (DSBs) by downregulating key members of the homologous recombination (HR) network. Furthermore, we showed that the induced expression of miR-449a was able to amplify the toxic effect of the PARP inhibitor Olaparib in cells with the pathogenic germline *BRAC1* variant. This could help to improve TNBC treatments or to define more efficient alternative therapies.

## 2. Results

### 2.1. MiR-449a Is Epigenetically Silenced in TNBC and Re-Expression Leads to Reduced Cell Viability and Induced Apoptosis in TNBC Cell Lines

**Figure 1:**
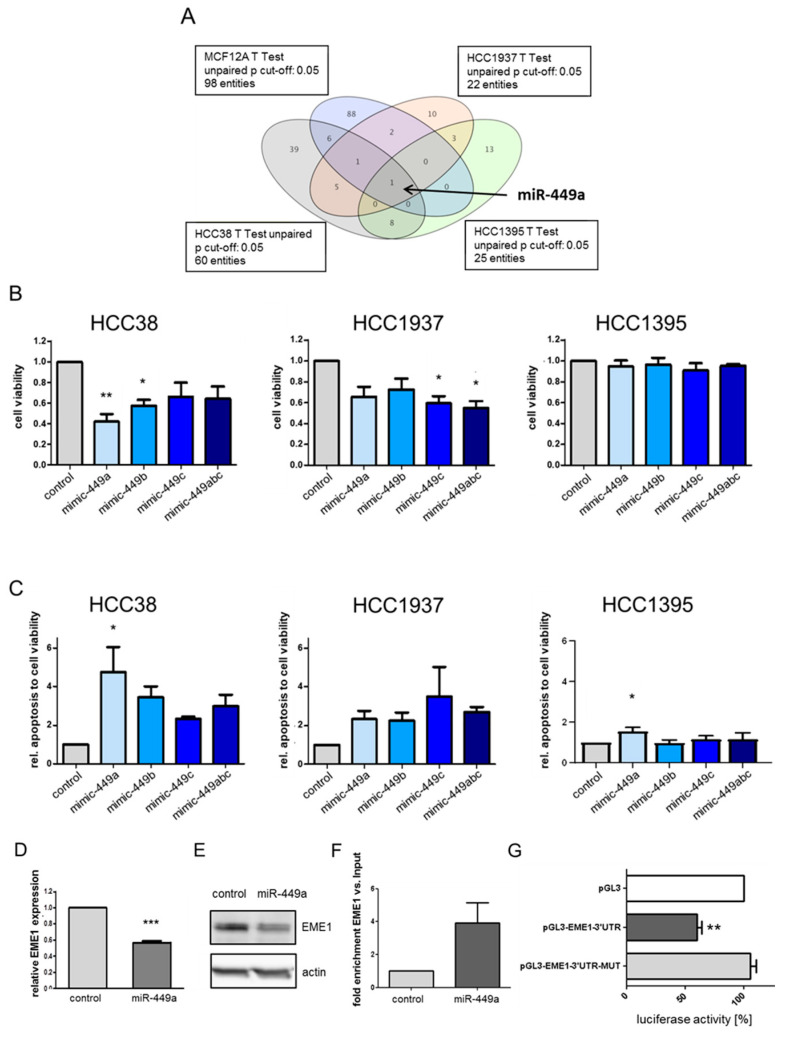
EME1 is a direct target of epigenetically regulated tumor suppressive miR-449a in TNBC cell lines. (**A**) Identification of significantly upregulated miRNAs after TSA treatment for 24 h by intersection using one-way ANOVA followed by Dunnett’s multiple comparison test. *p* < 0.1; FC ≥ 4. (**B**) Cell viability analyzed by WST-1 assay in TNBC cells transfected with miR-449-mimics normalized to control (Allstars Negative Control) transfected cells. (**C**) Apoptosis analyzed by caspase3/7 activity in TNBC cells transfected with miR-449-mimics normalized to cell viability and the control. Bar graphs show mean ± SD. N = 3, * *p* < 0.1, ** *p* < 0.01, *** *p* < 0.001. (**D**) Expression of EME1 measured by RT-PCR 48 h after transfection with miR-449a-mimics or control. (**E**) Expression of EME1 protein in HCC38 cells using Western blotting. Actin was used as a loading control on the same blot but on a different part of the gel. (**F**) Ago2 immunoprecipitation was performed and the enrichment of EME1 vs input was detected by RT-PCR. (**G**) Firefly luciferase activity measurements in HEK293 cells of miRNA-mimics in relation to renilla luciferase activity. 3′UTR: untranslated region of EME1, 3′UTR-MUT: untranslated region of EME1 with mutations in the predicted target site for miR-449a. Bar graphs show mean ± SD ** *p* < 0.01, *** *p* < 0.001, 2-tailed Student’s *t*-test for (**D**), one-way ANOVA/Dunnett’s multiple comparison test for (**B**,**C**).

Tumor suppressive miRNAs have been described to be epigenetically downregulated in breast cancer [11]. To identify epigenetically silenced microRNAs in TNBC, the TNBC cell lines HCC38, HCC1937, and HCC1937, as well as the immortalized breast cell line MCF12A were treated with TSA followed by microarray analyses, as we described previously [12]. This identified miR-449a as the strongest upregulated miRNA by comparing miRNA expressions that were at least 4-fold increased after TSA treatment in all analyzed cell lines (Figure 1A). Validation by quantitative PCR demonstrated that expression of miR449-family members miR-449a, miR-449b, and miR-449c was strongly induced by TSA between 10- and 20-fold in MCF12A, HCC1395 and HCC1937 cells, and between 20- and 45-fold in HCC38a cells (Figure S1A). The miR-449 family is located on cytogenetic band 5q11.2 and is encoded as a cluster within the host gene CDC20B (Figure S1B, [13]). Based on this, we performed chromatin immunoprecipitation experiments and indeed detected an increased acetylation at histones H3 (H3K9) and H4 (H4K5, H4K8, H4K12 and H4K16) in the promoter region of the host gene CDC20B (Figure S1C).

To investigate tumor suppressive effects of miR-449-family we performed transient transfections of TNBC cells with mimics of the different miR-449-family members. High expression of miR-449a, miR-449b and miR-449c resulted in reduced proliferation (Figure 1B) and increased apoptosis (Figure 1C). In HCC38 cells, the effect of miR-449a on proliferation and apoptosis was the most prominent compared to the other TNBC cell lines and other miR-449 family members. Cell viability decreased to 40% and apoptosis increased up to 4.7-fold. 

Therefore, we decided to focus on the miR-449 family member miR-449a. To analyze the expression of miR-449a in different breast cancer subtypes and normal breast-like tissue, we performed meta-analyses on miRNA expression using bc GenExMiner 4.5 [14], a statistical mining tool of published annotated breast cancer transcriptomic data from TCGA [15] and SCAN-B/GSE96058 [16] cohorts. The different breast cancer subtypes based on gene expression data defined by Sorlie et al. [17] showed no significantly different expression of miR-449a compared to normal breast-like tissue (Figure S2A). However, there were significant differences between the breast cancer subtypes. The expression of miR-449a was significantly reduced in basal-like breast cancer compared to luminal A breast cancer. Therefore, we analyzed the miR-449a expression in patients with TNBC and non-TNBC breast cancer. Here, we identified a significant reduction of miR-449a expression in patients with TNBC compared to non-TNBC (Figure S2B).

### 2.2. MiRNA-449a Deregulates Prominent Genes Involved in HDR and Directly Targets EME1

To provide a broader biological interpretation of miR-449a’s tumor suppressive function in TNBC, we next sought to identify miR-449a target genes by mRNA expression arrays. For this, we identified differentially expressed genes (*p* < 0.25, FC ≥ 1.5) after miR-449a mimic transfection and performed pathway analyses considering 290 WikiPathways with a cutoff of *p* < 0.01. We found that miR-449a significantly regulated several pathways that are related to DNA repair (e.g., DNA damage response, double-strand break repair, homologous recombination, Table S1). In particular, genes involved in the HDR pathway were deregulated (Figure S3). Interestingly, several additional online target identification tools including TargetScan, miRDB, and DIANA-microT-CDS also identified the endonuclease EME1 as a putative target gene of miR-449a. In line with this, EME1 expression on mRNA was significantly reduced to 58% (Figure 1D) as well as on protein level to 67% (Figure 1E, for quantification see Figure S4) after transfection of HCC38 cells with miR-449a-mimics. Downregulation of EME1 by miR-449a was validated in HCC1937 cells (Figure S5). To analyze if EME1 is a direct target of miR-449a, we performed RNA immunoprecipitation and found a significant enrichment (four-fold versus enrichment of input) of EME1 after transfection with miR-449a in comparison to cells transfected with control (Figure 1F). For luciferase reporter assays, we constructed vectors harboring the 3ʹUTR of EME1 including either the intact or a mutated binding site for the miR-449a (pGL3-EME1-3′UTR or pGL3 EME1-3′UTR-mut, respectively) downstream of a luciferase gene and co-transfected them with miR 449a into HEK293 cells. MiR-449a significantly reduced luciferase activity upon co-transfection with pGL3-EME1-3′UTR to 62% (Figure 1G). Co-transfection with pGL3-EME1-3′UTR-mut restored luciferase activity, confirming the predicted direct binding of miR-449a to EME1. Thus, we identified EME1 as a new target of miR-449a.

### 2.3. MiRNA-449a Leads to Incomplete Chromosome Segregation by Targeting EME1

EME1 in complex with MUS81 minimizes chromosome mis-segregation and non-disjunction [5]. The meta-analyses of publicly available datasets provided by the Gene Expression Profiling Interactive Analysis platform [18] and the bc-GenExMiner platform [14] revealed induced expression of EME1 in many tumor types as well as breast cancer (Figures S6 and S7). The expression of EME1 in TNBC was significantly higher compared to other breast cancer subtypes (non-TNBC) (Figure S8). In the dataset GSE58215 that provided miRNA expression as well as gene expression data in breast cancer, we observed no correlation between *miR-449a* and *EME1* expression (Figure S9).

**Figure 2:**
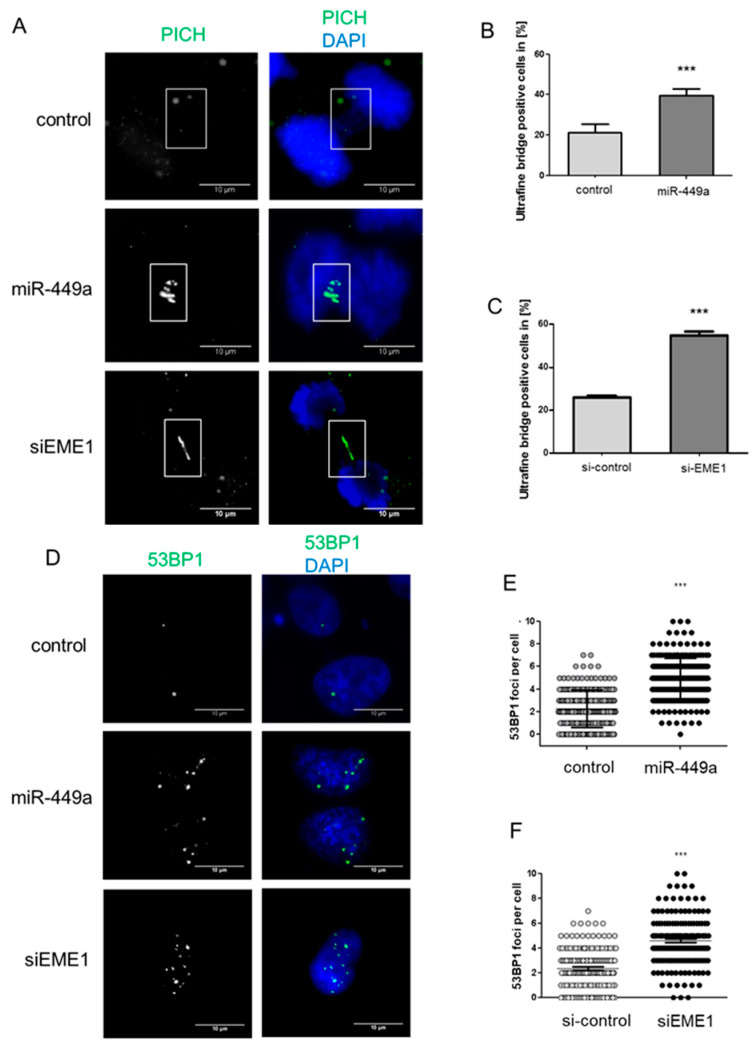

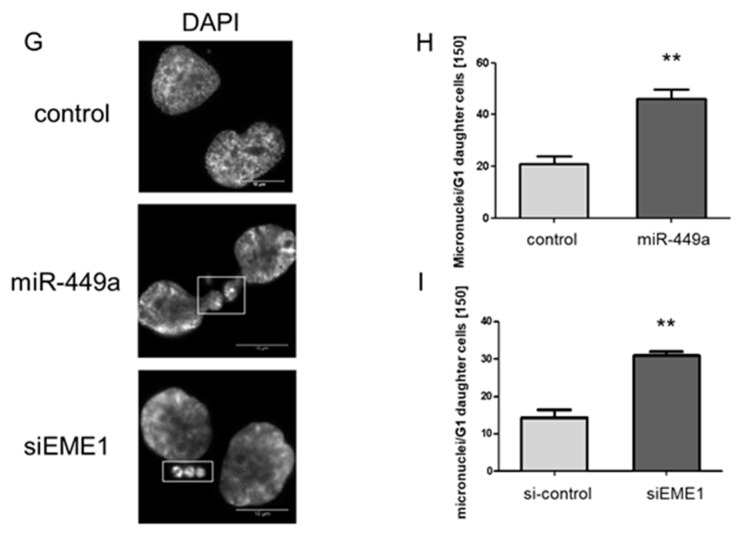
Induction of miR-449a expression and EME1 silencing both increase the frequency of ultrafine bridges, 53BP1 foci, and micronuclei. (**A**) Representative IF images and quantification (**B**,**C**) of PICH positive ultrafine bridges (green) in HCC38 cells transfected with miRNA mimics or control or with an EME1 siRNA. DNA was stained using DAPI (4′,6-Diamidin-2-phenylindol) (blue). N = 150. (**D**) Representative IF images and quantification (**E**,**F**) of 53BP1 positive foci (green) in HCC38 cells transfected with miRNA-mimics or control, or with an EME1 siRNA. DNA was stained using DAPI (blue). N = 150 (**G**) Representative images of micronuclei stained using DAPI and quantification (**H**,**I**). N = 150. Bar graphs show mean ± SEM. Error bars represent SEM. N = 3. ** *p* < 0.01, *** *p* < 0.001, 2-tailed Student’s *t*-test. Scale bars = 10 µm.

To assess the contribution of miR-449a in the segregation and disjunction of chromosomes, we examined whether the increased expression of miR-449a leads to chromosomal instability. The transfection of HCC38 cells with miRNA-449a-mimics resulted in a two-fold increase in the frequency of common fragile site-associated ultra-fine anaphase DNA bridges (22% in control, 40% in miR-449a-mimic transfected cells) (Figure 2A,B). This increase was comparable with frequencies observed after the siRNA-mediated knockdown of EME1 (26% in si-control, 53% in si-EME1 transfected cells) (Figure 2A,C). The Si-mediated knockdown of EME1 and all following genes was proven by Western blot (Figure S8). Furthermore, we detected a corresponding two-fold increase in the number of 53BP1 nuclear bodies that are known to be associated with mis-segregated CFSs [5,19] (Figure 2D–F) and a two-fold increase of micronuclei formation in G1 daughter cells after transfection with miR-449a-mimics or siEME1 (Figure 2G–I). Taken together, we propose that the increased expression of tumor suppressive miR-449a leads to defects in the segregation and disjunction of chromosomes, known drivers of chromosomal instability.

### 2.4. MiRNA-449a Reduces Homology-Directed Repair by Downregulation of E2F3 and Survivin

**Figure 3:**
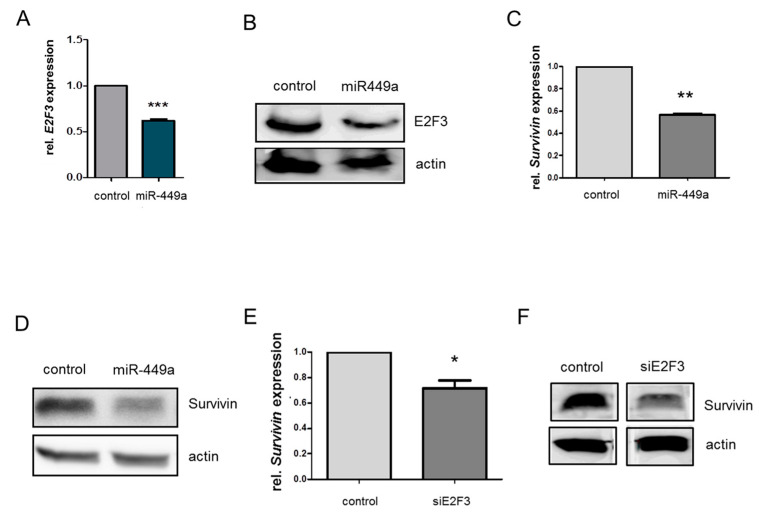
MiR-449a decreases expression of Survivin indirectly by targeting E2F3. (**A**) Expression of E2F3 mRNA measured by RT-PCR 48 h after transfection with miR-449a-mimics or control. (**B**) Expression of E2F3 protein using Western blotting in HCC38 cells. Actin was used as a loading control on the same blot but on a different part of the gel. (**C**) Expression of Survivin mRNA measured by RT-PCR 48 h after transfection with miR-449a-mimics or control on mRNA level. (**D**) Expression of Survivin protein using Western blot in HCC38 cells. Actin was used as a loading control on the same blot but on a different part of the gel. (**E**) Expression of Survivin mRNA measured by RT-PCR 48 h after transfection with siE2F3 or si-control. (**F**) Expression of Survivin protein using Western blot in HCC38 cells after transfection with siE2F3 or si-control. Actin was used as a loading control on the same blot but on a different part of the gel. Error bars represent SEM. N = 3. * *p* < 0.05, ** *p* < 0.01, *** *p* < 0.001, 2-tailed Student’s *t*-test.

Key players in DNA repair by homologous recombination such as BRCA2, RAD51, and BIRC5 coding for Survivin, were additionally identified as significantly deregulated upon miR-449a-mimic transfection using mRNA expression arrays. Thus, we aimed to identify target genes of miR-449a that could explain the downregulation of HDR genes upon miR-449a-mimic transfection. We observed the significantly reduced mRNA expression of a known miR-449a target, the E2F transcription factor, E2F3 [20], in mRNA expression arrays and validated the results by qPCR and Western blot (Figure 3A,B). The expression of mRNA was reduced to 54%. Protein levels were reduced less upon the transfection of miR-449a-mimics to 90% (Figure S4). The reduced expression on protein level was more obvious in the TNBC cell line HCC1937 (Figure S5). Furthermore, we detected reduced Survivin mRNA expression and decreased levels of Survivin protein upon transfection of miR-449a in HCC38 (Figure 3C,D, Figure S4) and in HCC1937 (Figure S5) as well as after silencing of E2F3 (Figure 3E,F, Figures S5 and S10). The reduced expression of Survivin upon silencing E2F3 is in line with studies that have described E2F3 as a transcription factor of Survivin [21]. Thus, the downregulation of Survivin by miR-449a can be in part explained by targeting E2F3.

**Figure 4:**
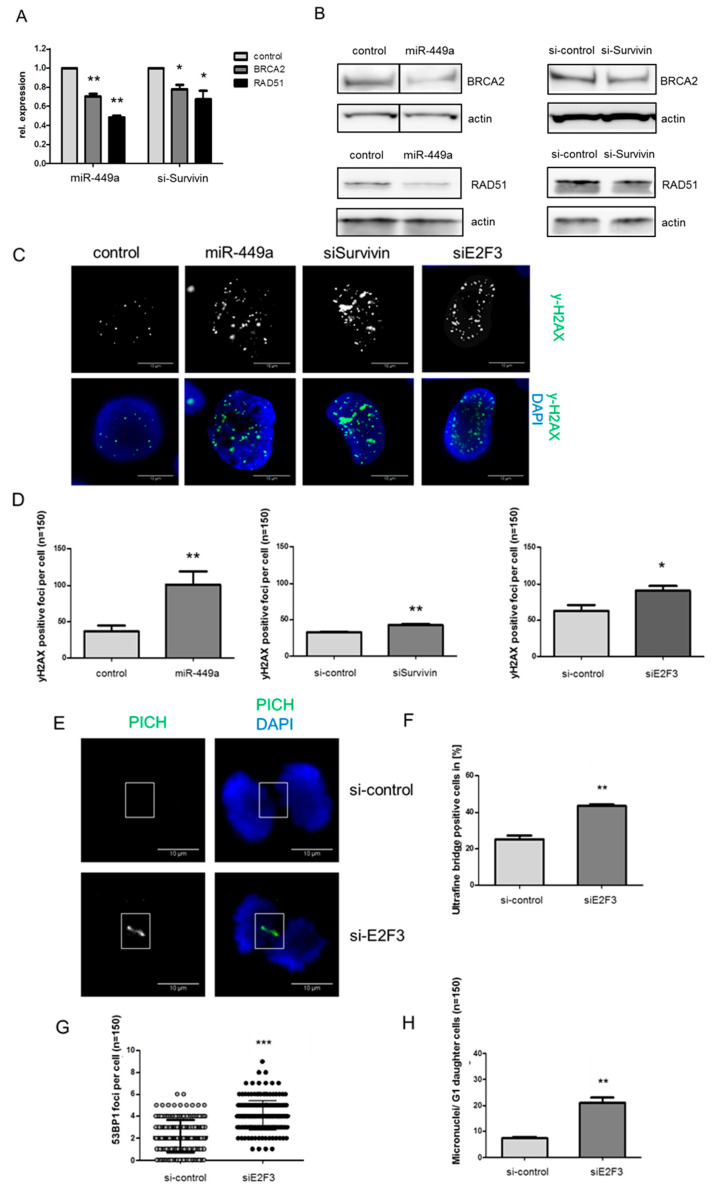
MiR-449a decreases expression of RAD51 and BRCA2 leading to increased yH2AX signals. (**A**) Expression of BRCA2 and RAD51 mRNA measured by RT-PCR 48 h after transfection with miR-449a-mimics/control or with siE2F3/si-control. (**B**) Expression of BRCA2 and RAD51 proteins using Western blotting in HCC38 cells. Actin was used as a loading control on the same blot but on a different part of the gel. (**C**) Representative IF images and quantification (**D**) of yH2AX positive foci (green) after transfection with miR-449a-mimics, siSurvivin or siE2F3 compared to control or si-control respectively. DNA was stained using DAPI (blue). (**E**) Representative IF images and quantification (**F**) of PICH positive ultrafine bridges (green) after transfection with siE2F3 or si-control in HCC38 cells. (**G**) Quantification of 53BP1 positive foci (green) or micronuclei (**H**) stained using DAPI. Error bars represent SEM. N = 3. * *p* < 0.05, ** *p* < 0.01, *** *p* < 0.001, 2-tailed Student’s *t*-test. Scale bars = 10 µm.

Survivin has been shown to downregulate members of the HR pathway such as BRCA2 and RAD51 in breast cancer [4]. We confirmed the downregulation of these genes in the TNBC cell line HCC38 by siRNA against Survivin on mRNA and protein level (Figure 4A,B and Figure S8). We then analyzed the effect of miR-449a on downstream targets of Survivin. Indeed, the mRNA expression of BRCA2 and RAD51 was reduced by 65% and 44%, respectively (Figure 4A). The protein expression of BRCA2 and RAD51 was also significantly reduced after the transfection of miR-449a-mimics (Figure 4B, Figure S4). In order to analyze whether the induced expression of miR-449a has an influence on DNA damage by targeting E2F3 directly and Survivin indirectly, we counted Ser139 phospho-H2AX (yH2AX) positive foci after miR-449a-mimic transfection. Indeed, we observed a 2.3-fold increase in yH2AX-signals in miR-449a-mimic transfected cells compared to control (Figure 4C,D). Silencing Survivin or E2F3 also led to significantly increased levels of yH2AX-foci (Figure 4C,D). 

Considering the major role of BRCA2 and RAD51 in chromosome segregation [19], we set out to assess the contribution of E2F3 in chromosome segregation. We observed that the reduced expression of E2F3 using siRNA (Figure S10) resulted in a 1.7-fold increase of ultra-fine anaphase DNA bridge frequency (Figure 4E,F), a 2-fold increase in 53BP1 foci, and the increased formation of micronuclei in G1 daughter cells (Figure 4G,H).

### 2.5. MiR-449a-Mediated Chromosomal Instability Increases the Toxic Effect of Olaparib in HCC1937 Cells

**Figure 5:**
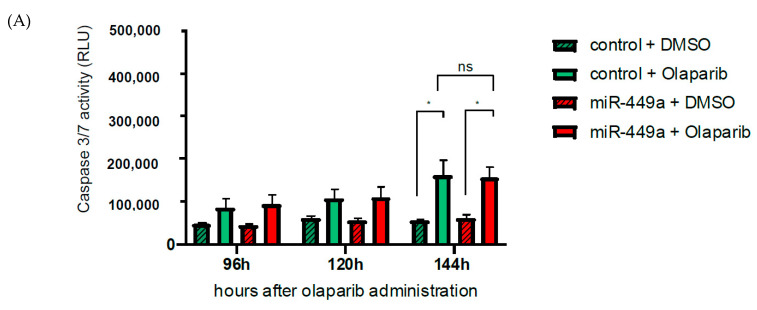

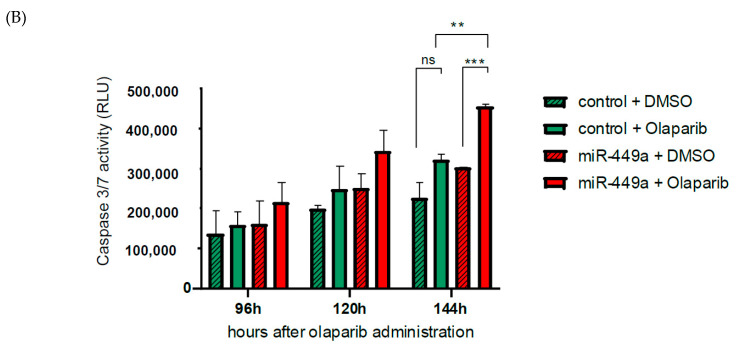
Induced expression of miR-449a in combination with Olaparib strongly decreased the amount of viable HCC1937 cells with a pathogenic variant in BRCA1. HCC38 (**A**) and HCC1937 (**B**) cells were treated with Olaparib (DMSO as control) and transfected with miR-449a (or control). Apoptosis analyzed by caspase3/7 activity in TNBC cells transfected with miR-449-mimics normalized to cell viability. Bar graphs show mean ± SD * *p* < 0.05, ** *p* < 0.01, *** *p* < 0.001, ns: not significant; 2-way Anova with Tukey’s multiple comparisons.

Based on our findings that induced expression of miRNA-449a resulted in a decrease of homology-directed repair and an increase in chromosome mis-segregation, we investigated whether a combined therapy with miR-449a-mimics and PARP inhibitors such as Olaparib could increase the level of apoptosis in TNBC cells and whether the effect is different in a background of genetic DNA repair deficiency. For this, we performed a transfection with miR-449a-mimics and the control in the TNBC cell line HCC38 and in HCC1937 during Olaparib treatment. To characterize the cell lines further, we sequenced the tumor and their corresponding lymphoblastic cell lines and analyzed 22 genes (ATM, BAP1, BARD1, BRCA1, BRCA2, BRIP1, CDH1, CHEK2, FANCA, FANCC, FANCM, MLH1, MSH2, MSH6, NBN, PALB2, PMS2, PTEN, RAD51C, RAD51D, STK11, TP53) known to be associated with hereditary breast and ovarian cancer (Table S2). In cell line HCC38, we detected no germline variant, but in HCC1937 we detected a pathogenic variant in BRCA1 (c.5266dup;p.Gln1756ProfsTer74; class 5; het). In cells with wt BRCA, we observed an increase in apoptotic cells by Olaparib treatment, but no additive effect of Olaparib treatment and miR-449a-mimic transfection (Figure 5A). However, we detected a stronger increase in apoptotic cells by Olaparib treatment in HCC1937 cells compared to HCC38 cells and an additive effect of Olaparib treatment and miR-449a-mimic transfection (Figure 5B). To sum up, we demonstrated that the induced expression of miR-449a was able to amplify the toxic effect of Olaparib in cells with the pathogenic germline BRAC1 variant.

## 3. Discussion

TNBC patients have relatively low survival rates as they often relapse within 1–3 years after diagnosis. 15–20% of TNBC patients harbor germline pathogenic variants in *BRCA1* and *BRCA2*, which makes them sensitive towards PARP inhibitors. Here, we demonstrate a new mechanism by which the induced expression of miR-449a strongly increased CIN, leading to apoptosis in TNBC cells. The induced expression of miR-449a resulted in chromosome mis-segregation and increased DNA-DSB. Furthermore, the transfection of mimic-449a amplified the apoptotic effect of the PARP inhibitor Olaparib in cells with the pathogenic germline BRAC1 variant. This makes miR-449a an interesting therapeutic target for TNBC.

We identified the endonuclease *EME1* as a new target of miR-449a. EME1 minimizes the mis-segregation and non-disjunction of chromosomes [5]. By targeting *EME1*, miR-449a plays a new role in disturbing chromosome segregation at sites of CFSs. Other studies have demonstrated that cells lacking the SLX1-SLX4-MUS81-EME1 complex exhibit defects in chromosome segregation, which lead to the transmission of extensive DNA damage to daughter cells [5,22]. To our knowledge, this is the first describing miRNA-guided regulation of *EME1* expression.

Notably, we deciphered the mechanism by which miR-449a inhibits the DNA repair machinery. The induced expression of miR-449a was able to disturb the HDR pathway by downregulating the expression of HDR key players such as *Survivin*, *BRCA2*, and *RAD51* in HCC38 cells. We identified miR-449a to downregulate the transcription factor *E2F3*, which in turn leads to the downregulation of *Survivin*. *E2F3* has been shown to be a direct target of miR-449a [23] and is a transcription factor for *Survivin* [24]. Survivin itself is known to regulate HR factors such as *BRCA2* and *RAD51* [4]. In line with this report, we detected a reduced expression of *BRCA2* and *RAD51* after miR-449a-mimic transfection and silencing of *Survivin*. “We propose a model in which induced expression of miR-449a downregulates E2F3, and hence Survivin, which subsequently leads to decreased expression of BRCA2 and RAD51 resulting in inhibition of DNA repair. This hypothesis should be supported by future experiments with different techniques such as rescue experiments for each member of this signal cascade.”

Ultrafine anaphase bridges are markers of defective chromosome segregation that are thought to form when topological DNA entanglements between two chromatids are not resolved prior to anaphase onset. Here, we observed an increase in ultrafine anaphase DNA bridges upon silencing *E2F3*. Recently, *BRCA2* and *RAD51* have been described to contribute to replicative stress at CFSs [11]. Likewise, another study observed an increase in ultrafine DNA bridges in embryonic stem cells deficient for *BRCA1/2* or with dominant negative *Rad51-K133R/A* mutations [13]. We demonstrated that the miR-449a-mediated downregulation of *E2F3* results in the reduced expression of *BRCA2* and *RAD51* and propose that this results in a failure to disjoin sister chromatids. To sum up, we observed a novel tumor suppressive role of miR-449a by inhibiting DNA-DSB repair and effective chromosome segregation, resulting in massive CIN in TNBC cells. 

Different pathways of chromosomal stability are affected by miR-449a. Since the transfection of miR-449a-mimics addresses all these pathways simultaneously, the different effects sum up to a substantial increase in chromosomal instability and cell death. Recently, the PARP inhibitor Olaparib has been evaluated as a promising anti-cancer agent in women with germline pathogenic variants in *BRCA1* or *BRCA2* [25,26]. Here, we demonstrated that the induced expression of miRNA-449a enhanced the effect of Olaparib significantly. Marijon et al., have described that combined therapy of HDACi and Olaparib led to the inhibition of proliferation in HCC1937 cells [27]. This fits very well with our results, since the expression of miR-449a was induced by HDACi in our study. Therefore, we propose that miRNA-449a would be a suitable candidate for combination therapies with PARP inhibitors in TNBC. Based on our findings, a combined treatment of miRNA-449a-mimics and Olaparib might prove to be beneficial, particularly in women with pathogenic germline variants in *BRCA1*.

## 4. Materials and Methods

Additional methods and details are available in the supporting information.

Cell culture und transfection: TNBC cell lines HCC38 (RRID:CVCL_1267), HCC1395 (RRID:CVCL_1249), HCC1937 (RRID:CVCL_0290), their corresponding lymphoblastic cell lines HCC38 BL (RRID:CRL_2346) and HCC1937 BL (RRID:CRL_2337), and immortalized normal breast cell line MCF12A (RRID:CVCL_3744) (all obtained from American Type Culture Collection (ATCC), Manassas, VA, USA) were cultured as recommended by ATCC. The cell lines were bought within the last three years and were authenticated by STR analyses in March 2022. Cells were cultivated no longer than 4 weeks. Mycoplasma tests (Life Technologies, Carlsbad, CA, USA) were performed after arrival of cells from ATCC and cells were immediately stored in liquid nitrogen. All experiments were performed with mycoplasma-free cells. For treatment with TSA (100 ng/mL) or ethanol vehicle as a control, the medium was renewed every 12 h. Cells were transfected with 5 nM Silencer Select siRNA-pools (Life Technologies, Carlsbad, CA, USA) or 50 nM miRNA-mimics (Qiagen, Hilden, Germany) using HiPerFect Transfection Reagent (Qiagen) and Allstars Negative Control (Qiagen), hereafter referred to as control. 

Proliferation and apoptosis assay: Cell viability and apoptosis were measured in triplicate using the WST-1 Proliferation Reagent (Roche, Basel, Switzerland) and the Caspase3/7 Glo Assay (Promega, Madison, WI, USA), respectively. 

Analysis of mRNA, miRNA, and protein expression: Total RNA, including miRNAs, was isolated using the Qiazol Lysis Reagent and the miRNeasy Mini Kit (both Qiagen). Expression of mRNA and miRNA was measured in triplicate by quantitative real-time PCR (qRT-PCR) using Taqman Gene Expression Assays and Taqman MicroRNA Assays (both Life Technologies). Global miRNA and mRNA expression profiling was performed using two microarrays per condition. SurePrint G3 Human miRNA release 21 Microarray Kit, 8x60K was used for miRNA expression profiling and the SurePrint G3 Human Gene Expression v3 8x60K Microarray Kit (both Agilent, Santa Clara, CA, USA) was used for mRNA expression profiling according to manufacturer’s instructions. In brief, RNA of three biological replicates per microarray was pooled and labeled using the Low Input Quick Amp Labeling Kit (Agilent). After hybridization, washing, and scanning of the arrays signal intensities were extracted with Feature Extraction software (Agilent) and normalized by percentile shift to the 75th percentile. Significantly upregulated miRNAs after TSA treatment were identified using one-way ANOVA followed by Dunnett’s multiple comparison test. *p* < 0.1; FC ≥ 4. Significantly upregulated putative target genes after miR-449a transfection were identified using one-way ANOVA followed by Dunnett’s multiple comparison test. *p* < 0.25; FC ≥ 1.5. Pathway analyses of putative target genes were performed considering 290 WikiPathways with a cutoff of *p* < 0.01. All microarray analyses were performed with the GeneSpring GX software (Agilent). For protein analysis, whole cell lysates were prepared with RIPA buffer employing equal numbers of cells. Lysates were sonicated three times for 10 sec with 1 min breaks on ice. Cell debris was removed by centrifugation at 4 °C and 16,200× *g* for 20 min. Proteins were separated on 12% SDS polyacrylamide gels and transferred to nitrocellulose membranes. Membranes were blocked with 5% milk in PBS including 0.05% Tween-20 (PBS-T) for 1 h. Membranes were incubated with primary antibodies overnight at 4 °C. After washing with PBS-T three times for 5 min, membranes were incubated with HRP-conjugated secondary antibodies for 1 h. After washing the membranes with PBS T three times for 5 min, protein bands were detected with SuperSignal West Femto Maximum Sensitivity Substrate (Life Technologies) using the LAS-3000 Imaging System (Fujifilm, Tokyo, Japan). Quantification of protein expression was analyzed by Image Studio Lite Version 5.2 (Bad Homburg, Germany).

Luciferase reporter assay: Luciferase reporter vectors (pGL3-vector (Promega)) were constructed containing the 3′-UTR of EME1 fragments called gBlocks Gene Fragments (Integrated DNA Technologies, Leuven, Belgium) with intact binding sites for miR-449a (pGL3-EME1) or with mutated binding sites (pGL3-mutEME1). For transfection, 8000 HEK293 cells were seeded in white 96-well plates. The next day, combinations of 20 nM miRNA-mimics and 25 ng luciferase reporter vectors were transfected in triplicate using Lipofectamine 2000 (Life Technologies). For normalization, pGL4.70 (Promega, Madison, WI, USA) with an EF1α promoter inserted at the XhoI restriction site upstream of renilla luciferase was co-transfected in all conditions. Luciferase activities were measured with the DualGlo Luciferase Assay System (Promega) 24 h after transfection.

RNA immunoprecipitation: In brief, miR-449a versus control transfected HCC38 cells were lysed in 100 µL polysomal lysis buffer. Then, dynabeads (Invitrogen, Carlsbad, CA, USA) were prepared and incubated with control IgG or Ago2 antibody. To control and monitor successful immunoprecipitation (IP), an aliquot of beads was taken and Ago2 pulldown was analysed by Western blot. After IP, RNA was isolated by the use of Trizol (Sigma-Aldrich, Taufkirchen, Germany). Isolated RNA from Ago2-IP was subjected to qPCR for validation. 

Cell synchronization: Asynchronously growing HCC38 cells were synchronized in late G2 phase of the cell cycle by incubation with 9 μM RO3306 (217699; Millipore) for 24 h. Cells synchronized in G2 were subsequently washed with 1× PBS for 5 min and allowed to progress for a further 40 min into prometaphase before mitotic shake off. For collection of anaphase cells or early G1 daughter cells, pelleted prometaphase cells were re-seeded onto glass coverslips (Sigma-Aldrich) and were incubated at 37 °C in an atmosphere of 5% CO_2_ and allowed to progress into anaphase for a further 40 min or into the subsequent G1 phase for a further 140 min. 

Immunofluorescence microscopy: Cells for immunofluorescence analysis were fixed and permeabilized for 20 min in PTEMF buffer at room temperature. Fixed samples were blocked for at least 1 h at room temperature using 3% BSA (Sigma Aldrich) in PBS-T (PBS containing 0.5% Triton X-100 (Sigma-Aldrich)). Samples fixed on poly-lysine glass slides were incubated with primary antibodies overnight at 4 °C followed by 4 washes of 15 min each using PBST supplemented with 3% BSA (Sigma-Aldrich). Samples were then incubated with secondary antibodies for 90 min, followed by 4 washes of 15 min using PBS-T supplemented with 3% BSA. Air-dried slides or coverslips were mounted using Vectashield mounting medium (Vector Laboratories, Burlingame, CA, USA) and were analyzed using microscopy.

Olaparib treatment: On day one, 4400 TNBC cells per well were seeded in a 96-well plate. On day two, (after 24 h) 10 µM Olaparib (Selleck Chemicals GmbH, Houston, TX, USA) or DMSO as a control were added per well. On day four, the cells were transfected with 50 nM miR449a-mimics or Allstars Negative Control. Furthermore, 10 µM Olaparib or DMSO were added. On day five, day six, and day seven, 10 µM Olaparib or DMSO were added. Measurements were performed 72 h, 96 h, 120 h and 144 h after first Olaparib treatment. 

Statistics: Data are represented as mean ± SD of at least three independent experiments unless stated otherwise. In figure legends, n represents the number of independent experiments. Statistical significance was determined by a 2-tailed Student’s *t* test, by a one-way ANOVA followed by Dunnett’s multiple comparison test. All statistical analyses were completed with the GraphPad Prism software (GraphPad Software, La Jolla, CA, USA). The experimenters were not blind to group assignment but did not know the outcome assessment. 

Availability of Data and Materials: The datasets generated and analyzed during the current study will all be deposited in publicly available repositories during the reviewing process. 

## Data Availability

All data will be made available in case of publishing.

## Supplementary Materials

The following supporting information can be downloaded at: https://www.mdpi.com/article/10.3390/ijms23095131/s1.

## References

[1] Stevens K.N., Vachon C.M., Couch F.J. (2013). Genetic Susceptibility to Triple-Negative Breast Cancer. Cancer Res..

[2] Liedtke C., Mazouni C., Hess K.R., André F., Tordai A., Mejia J.A., Symmans W.F., Gonzalez-Angulo A.M., Hennessy B., Green M. (2008). Response to Neoadjuvant Therapy and Long-Term Survival in Patients With Triple-Negative Breast Cancer. J. Clin. Oncol..

[3] Birnbaum M.J., Clem R.J., Miller L.K. (1994). An apoptosis-inhibiting gene from a nuclear polyhedrosis virus encoding a polypeptide with Cys/His sequence motifs. J. Virol..

[4] Véquaud E., Desplanques G., Jézéquel P., Juin P., Barille-Nion S. (2015). Survivin contributes to DNA repair by homologous recombination in breast cancer cells. Breast Cancer Res. Treat..

[5] Minocherhomji S., Ying S., Bjerregaard V.A., Bursomanno S., Aleliunaite A., Wu W., Mankouri H., Shen H., Liu Y., Hickson I.D. (2015). Replication stress activates DNA repair synthesis in mitosis. Nature.

[6] Weinandy A., Piroth M.D., Goswami A., Nolte K., Sellhaus B., Gerardo-Nava J., Eble M., Weinandy S., Cornelissen C., Clusmann H. (2014). Cetuximab Induces Eme1-Mediated DNA Repair: A Novel Mechanism for Cetuximab Resistance. Neoplasia.

[7] Bartel D.P. (2004). MicroRNAs: Genomics, Biogenesis, Mechanism, and Function. Cell.

[8] Naorem L.D., Muthaiyan M., Venkatesan A. (2018). Identification of dysregulated miRNAs in triple negative breast cancer: A meta-analysis approach. J. Cell. Physiol..

[9] Buurman R., Gürlevik E., Schäffer V., Eilers M., Sandbothe M., Kreipe H., Wilkens L., Schlegelberger B., Kühnel F., Skawran B. (2012). Histone Deacetylases Activate Hepatocyte Growth Factor Signaling by Repressing MicroRNA-449 in Hepatocellular Carcinoma Cells. Gastroenterology.

[10] Sandbothe M., Buurman R., Reich N., Greiwe L., Vajen B., Gurlevik E., Schäffer V., Eilers M., Kühnel F., Vaquero A. (2017). The microRNA-449 family inhibits TGF-beta-mediated liver cancer cell migration by targeting SOX4. J. Hepatol..

[11] Ali S.R., Humphreys K.J., McKinnon R., Michael M.Z. (2015). Impact of Histone Deacetylase Inhibitors on microRNA Expression and Cancer Therapy: A Review. Drug Dev. Res..

[12] Vajen B., Greiwe L., Schäffer V., Eilers M., Huge N., Stalke A., Schlegelberger B., Illig T., Skawran B. (2021). MicroRNA -192-5p inhibits migration of triple negative breast cancer cells and directly regulates Rho GTPase activating protein 19. Genes Chromosom. Cancer.

[13] Kent W.J., Sugnet C.W., Furey T.S., Roskin K.M., Pringle T.H., Zahler A.M., Haussler D. (2002). The Human Genome Browser at UCSC. Genome Res..

[14] Jézéquel P., Gouraud W., Ben Azzouz F., Guérin-Charbonnel C., Juin P.P., Lasla H., Campone M. (2021). bc-GenExMiner 4.5: New mining module computes breast cancer differential gene expression analyses. Database.

[15] National Cancer Institute The Cancer Genome Atlas Program (TCGA). https://www.cancer.gov/tcga.

[16] Saal L.H., Vallon-Christersson J., Häkkinen J., Hegardt C., Grabau D., Winter C., Brueffer C., Tang M.-H.E., Reuterswärd C., Schulz R. (2015). The Sweden Cancerome Analysis Network-Breast (SCAN-B) Initiative: A large-scale multicenter infrastructure towards implementation of breast cancer genomic analyses in the clinical routine. Genome Med..

[17] Sørlie T., Tibshirani R., Parker J., Hastie T., Marron J.S., Nobel A., Deng S., Johnsen H., Pesich R., Geisler S. (2003). Repeated observation of breast tumor subtypes in independent gene expression data sets. Proc. Natl. Acad. Sci. USA.

[18] Tang Z., Li C., Kang B., Gao G., Li C., Zhang Z. (2017). GEPIA: A web server for cancer and normal gene expression profiling and interactive analyses. Nucleic Acids Res..

[19] Bhowmick R., Minocherhomji S., Hickson I.D. (2016). RAD52 Facilitates Mitotic DNA Synthesis Following Replication Stress. Mol. Cell.

[20] Fang Y., Gu X.-D., Li Z., Xiang J., Chen Z. (2013). miR-449b inhibits the proliferation of SW1116 colon cancer stem cells through downregulation of CCND1 and E2F3 expression. Oncol. Rep..

[21] Laulier C., Cheng A., Stark J.M. (2011). The relative efficiency of homology-directed repair has distinct effects on proper anaphase chromosome separation. Nucleic Acids Res..

[22] Sarbajna S., Davies D., West S.C. (2014). Roles of SLX1–SLX4, MUS81–EME1, and GEN1 in avoiding genome instability and mitotic catastrophe. Genes Dev..

[23] Ren X.-S., Yin M.-H., Zhang X., Wang Z., Feng S.-P., Wang G.-X., Luo Y.-J., Liang P.-Z., Yang X.-Q., He J.-X. (2014). Tumor-suppressive microRNA-449a induces growth arrest and senescence by targeting E2F3 in human lung cancer cells. Cancer Lett..

[24] Geng D., Song X., Ning F., Song Q., Yin H. (2015). MiR-34a Inhibits Viability and Invasion of Human Papillomavirus–Positive Cervical Cancer Cells by Targeting E2F3 and Regulating Survivin. Int. J. Gynecol. Cancer.

[25] Fong P.C., Boss D.S., Yap T.A., Tutt A., Wu P., Mergui-Roelvink M., Mortiner P., Swaisland H., Lau A., Connor M.J. (2009). Inhibition of poly(ADP-ribose) polymerase in tumors from BRCA mutation carriers. N. Engl. J. Med..

[26] Tutt A., Robson M., Garber J.E., Domchek S.M., Audeh M.W., Weitzel J.N., Friedlander M., Arun B., Loman N., Schmutzler R.K. (2010). Oral poly(ADP-ribose) polymerase inhibitor olaparib in patients with BRCA1 or BRCA2 mutations and advanced breast cancer: A proof-of-concept trial. Lancet.

[27] Marijon H., Lee D.H., Ding L.-W., Sun H., Gery S., de Gramont A., Koeffler H.P. (2018). Co-targeting poly(ADP-ribose) polymerase (PARP) and histone deacetylase (HDAC) in triple-negative breast cancer: Higher synergism in BRCA mutated cells. Biomed. Pharmacother..

