# Supplementary material for: MicroRNA-449a Inhibits Triple Negative Breast Cancer by Disturbing DNA Repair and Chromatid Separation
Source: Int J Mol Sci. 2022 May 4;23(9):5131. doi: 10.3390/ijms23095131 (PMC9102308; doi:10.3390/ijms23095131)
Supplement: Supplementary file 1 [file ijms-23-05131-s001.zip › ijms-1684607 - supplementary.pdf]

Supportive Information

Supplementary Tables

Table S1: Pathways regulated by miR-449a.

48 h after transfection of HCC38 cell with miR-449a-mimics, global gene expression was measured using mRNA microarrays. Pathway analyses of putative target genes (P< 0.25, FC< 1.5) were performed considering 290 WikiPathways with a cutoff of P<0.01.

Pathway	p-value	Matched Entities	Pathway Entities of Experiment Type
Hs_miRNA_Regulation_of_DNA_Damage_Response_WP1530_79564	0	17	98
Hs_Retinoblastoma_(RB)_in_Cancer_WP2446_80173	0	24	87
Hs_Mitotic_Metaphase_and_Anaphase_WP2757_77009	0	33	153
Hs_Retinoblastoma_(RB)_in_Cancer_WP2446_80174	0	24	87
Hs_Mitotic_Prometaphase_WP2652_76819	0	34	98
Hs_Cell_Cycle_WP179_70629	0	23	103
Hs_DNA_Damage_Response_WP707_79975	5,39E-11	16	68
Hs_Nucleosome_assembly_WP1874_76826	5,44E-10	10	22
Hs_ATM_Signaling_Pathway_WP2516_79964	1,65E-08	11	41
Hs_Mitotic_G1-G1-S_phases_WP1858_76928	2,18E-08	18	120
Hs_G1_to_S_cell_cycle_control_WP45_80001	9,69E-07	12	68
Hs_Fanconi_Anemia_pathway_WP1816_76931	1,36E-06	7	22
Hs_Mitotic_G2-G2-M_phases_WP1859_77022	2,46E-06	13	89
Hs_Kinesins_WP1842_76861	7,55E-06	6	18
Hs_Integrated_Cancer_pathway_WP1971_71249	1,07E-05	8	36
Hs_Gastric_Cancer_Network_1_WP2361_79693	1,67E-05	7	29
Hs_miR-targeted_genes_in_lymphocytes_-_TarBase_WP2004_79972	2,94E-05	29	495
Hs_Integrated_Pancreatic_Cancer_Pathway_WP2377_71228	4,60E-05	18	200
Hs_S_Phase_WP2772_77049	4,87E-05	13	116
Hs_miR-targeted_genes_in_muscle_cell_-_TarBase_WP2005_79979	1,17E-04	24	409
Hs_Meiotic_Recombination_WP2698_76904	2,11E-04	6	29
Hs_DNA_Replication_WP466_79981	2,62E-04	7	42
Hs_miR-targeted_genes_in_epithelium_-_TarBase_WP2002_78530	2,81E-04	20	345
Hs_Double-Strand_Break_Repair_WP1807_76953	3,61E-04	5	21
Hs_Extracellular_matrix_organization_WP2703_76914	3,73E-04	8	58
Hs_Assembly_of_collagen_fibrils_and_other_multimeric_structures_WP2798_77089	3,73E-04	6	33
Hs_Homologous_recombination_WP186_68935	5,01E-04	4	13
Hs_Integrated_Breast_Cancer_Pathway_WP1984_79991	6,65E-04	14	168
Hs_Cell_Cycle_Checkpoints_WP1775_76816	6,66E-04	11	115
Hs_Visual_phototransduction_WP2776_77056	8,13E-04	8	67
Hs_Metastatic_brain_tumor_WP2249_76471	9,14E-04	3	27
Hs_G13_Signaling_Pathway_WP524_72112	9,72E-04	6	38
Hs_Apoptosis_Modulation_and_Signaling_WP1772_80152	0,002058257	9	95
Hs_G_Protein_Signaling_Pathways_WP35_79952	0,002058257	9	92
Hs_Gastric_cancer_network_2_WP2363_76329	0,002338787	5	32
Hs_MHC_class_II_antigen_presentation_WP2679_76872	0,002458171	8	90
Hs_Signalling_by_NGF_WP1976_76994	0,002993401	6	50
Hs_Meiotic_Synapsis_WP2731_76957	0,003106187	5	35
Hs_Fluoropyrimidine_Activity_WP1601_80157	0,003106187	5	34
Hs_Nucleotide_Excision_Repair_WP1980_76859	0,003702206	6	49
Hs_Wnt_Signaling_Pathway_and_Pluripotency_WP399_79474	0,003881133	9	101
Hs_Interleukin-1_signaling_WP1839_76943	0,004575197	5	38
Hs_Metapathway_biotransformation_WP702_73516	0,00528001	12	188
Hs_Signal_Transduction_of_S1P_Receptor_WP26_79950	0,006621917	4	25
Hs_Mitotic_Prophase_WP2654_76823	0,007223194	5	44
Hs_Synthesis_of_DNA_WP1925_76968	0,007295437	8	94
Hs_Prostate_Cancer_WP2263_79838	0,00759918	9	116
Hs_Glycosaminoglycan_metabolism_WP2743_76981	0,008023065	5	41
Hs_Mitotic_Telophase-Cytokinesis_WP2765_77030	0,008094141	3	14
Hs_RNA_interference_WP2805_78493	0,008806697	2	5
Hs_Glycerophospholipid_biosynthesis_WP2740_76974	0,009224827	6	59
Hs_APC-C-mediated_degradation_of_cell_cycle_proteins_WP1782_77060	0,009952608	7	80

Table S2: Sequencing analyses of TNBC cell lines HCC38 and HCC1937 as well as their corresponding lymphoblastic cell line

Twenty-two genes known to be associated with Hereditary Breast and Ovarian Cancer (*ATM, BAP1, BARD1, BRCA1, BRCA2, BRIP1, CDH1, CHEK2, FANCA, FANCC, FANCM, MLH1, MSH2, MSH6, NBN, PALB2, PMS2, PTEN, RAD51C, RAD51D, STK11, TP53*) were sequenced. The target region was HE_HBOC_v03 with 408 regions and 89979 bases.

**Cell line**	**Variants in lymphoblastic cell line**	**Variants in tumor cell line**
HCC38		TP53;c.818G>T;p.Arg273Leu;class 5 variant; homozygous
HCC1937	BRCA1;c.5266dup;p.Gln1756ProfsTer74; class 5 variant; heterozygous	BRCA1;c.5266dup;p.Gln1756ProfsTer74; class 5 variant; heterozygous
		TP53,c.916C>T;p.Arg306Ter;class 5, homozygous

Supplementary figures

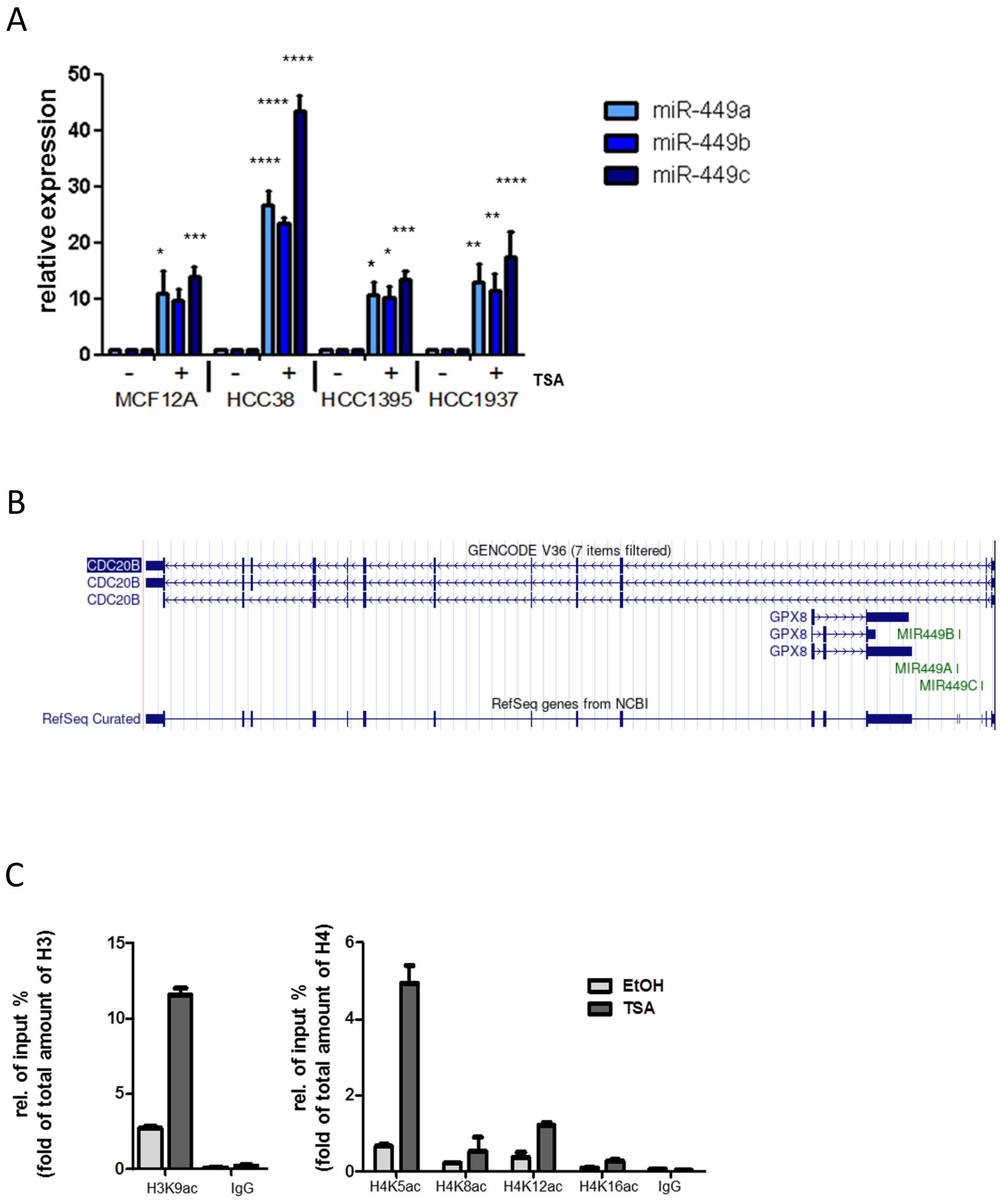

Figure S1: Induction of miR-449a expression by acetylation within the promoter region of host gene *CDC20B*

(**A**) Gene expression of miR-449a, miR-449b and miR-449c after 24 h stimulation with TSA normalized to ethanol treated cells. (**B**) The human genome browser at UCSC shows that miR-449-family is encoded as a cluster within their host gene *CDC20B* and located within cytogenetic band 5q11.2 (13). (**C**) ChIP of histone acetylation levels in the *CDC20B* promoter in HLE cells after TSA treatment in relation to the total amount of H3 or H4. * *P* < 0.1, ** *P* < 0.01, *** *P* < 0.001, **** *P* < 0.0001.

A

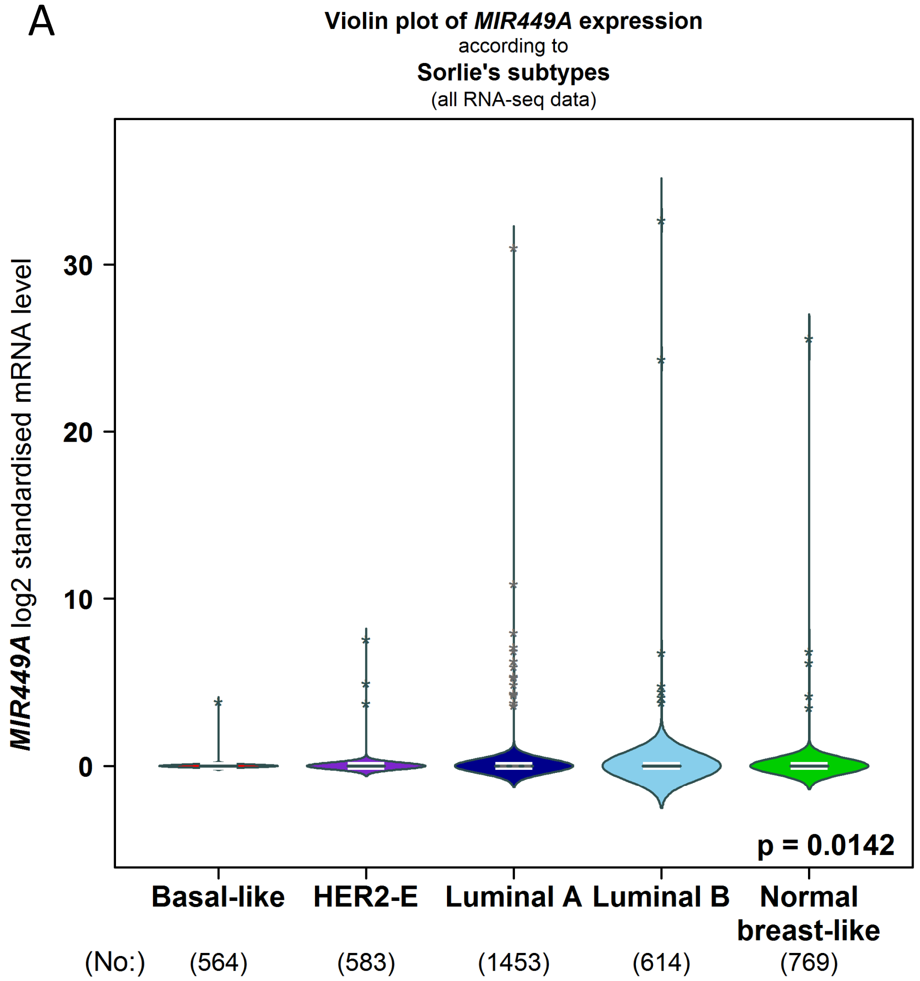

p(Welch) = 0.0142

Dunnett-Tukey-Kramer's test:

gene-expression comparisons			p-value
Luminal A	>	Basal-like	< 0.10
HER2-E	=	Basal-like	> 0.10
Luminal A	=	HER2-E	> 0.10
Luminal B	=	Basal-like	> 0.10
Luminal B	=	HER2-E	> 0.10
Luminal B	=	Luminal A	> 0.10
Normal breast-like	=	Basal-like	> 0.10
Normal breast-like	=	HER2-E	> 0.10
Normal breast-like	=	Luminal A	> 0.10
Normal breast-like	=	Luminal B	> 0.10

B

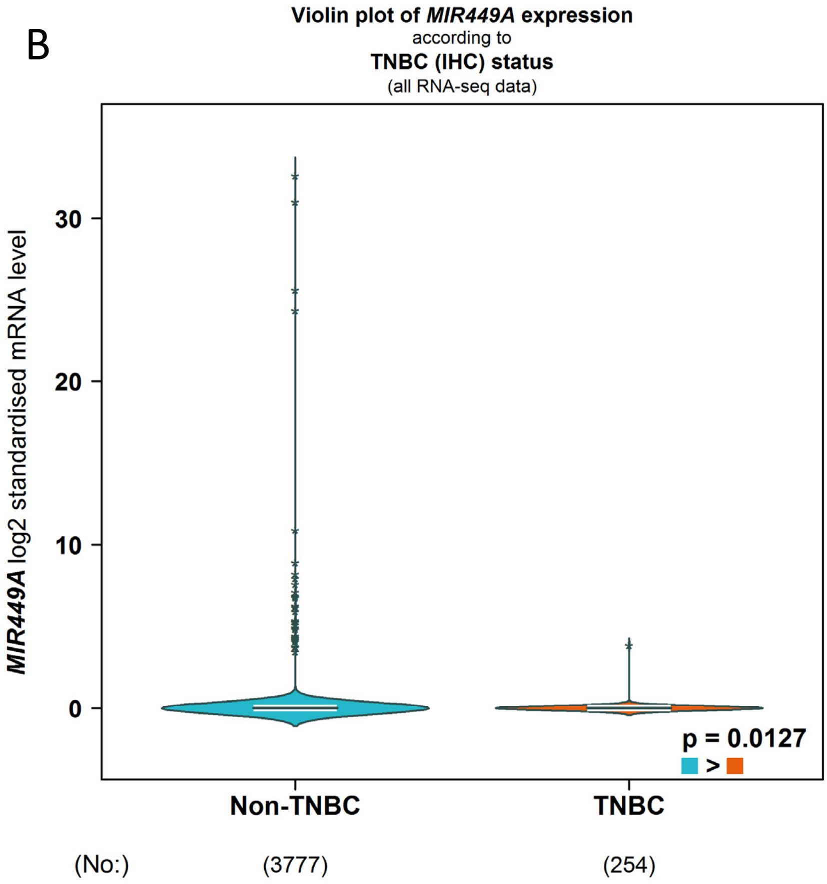

Figure S2: Meta-analyses on miR-449a expression in different breast cancer subtypes. (**A**) Violin plot of miR-449a expression according to Sorlie´s subtypes. Analyses were performed by bc-GenExMiner (14). The different breast cancer subtypes were defined by different expression patterns by Sorlie et al. (17). (**B**) Violin plot of miR-449a expression according to TNBC status. Statistical test: Welch test and Dunnett-Tukey-Kramer`s test.

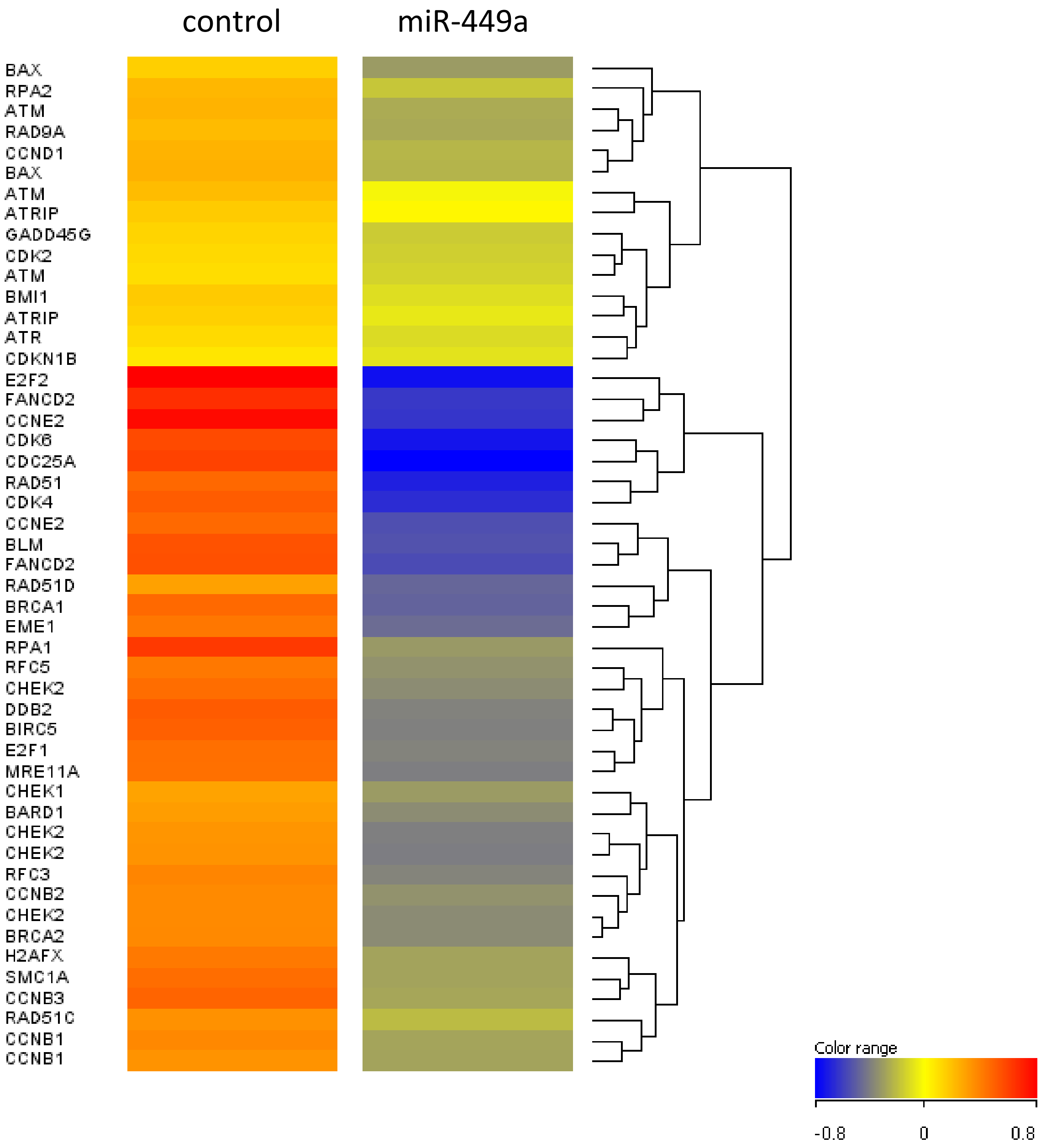

Figure S3: MiR-449a reduces expression of homology directed repair genes

Identification of differently expressed genes by global mRNA expression profiling after transfection of HCC38 cells with miR-449a mimics. Differentially expressed genes were hierarchically clustered with Euclidean distance metrics and Ward’s linkage rule. n=4

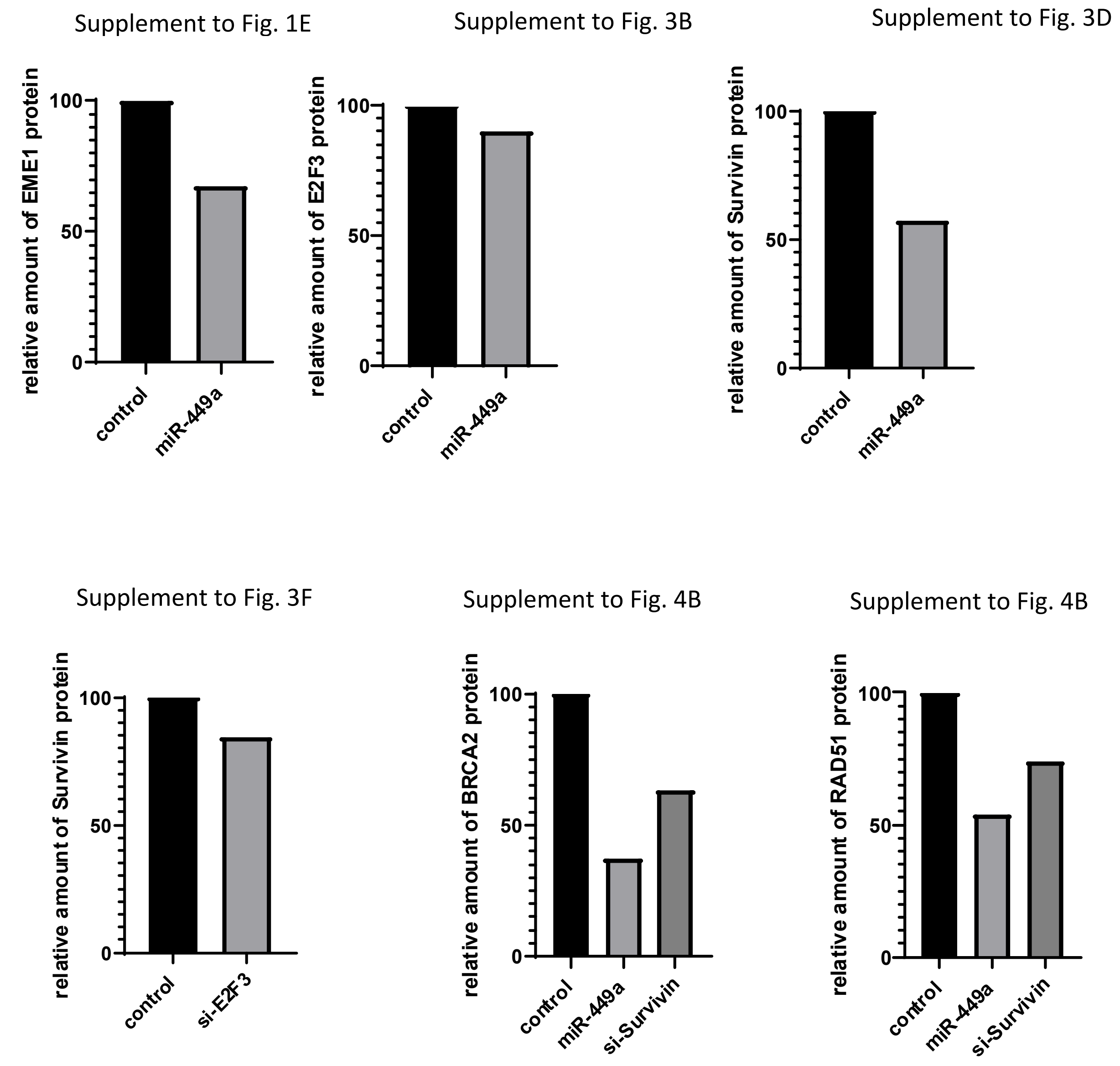

Figure S4: Quantification of Western Blots

Western Blots shown in Figures 1E, 3B-F and 4B were quantified by Image Studio Lite Version 5.2.

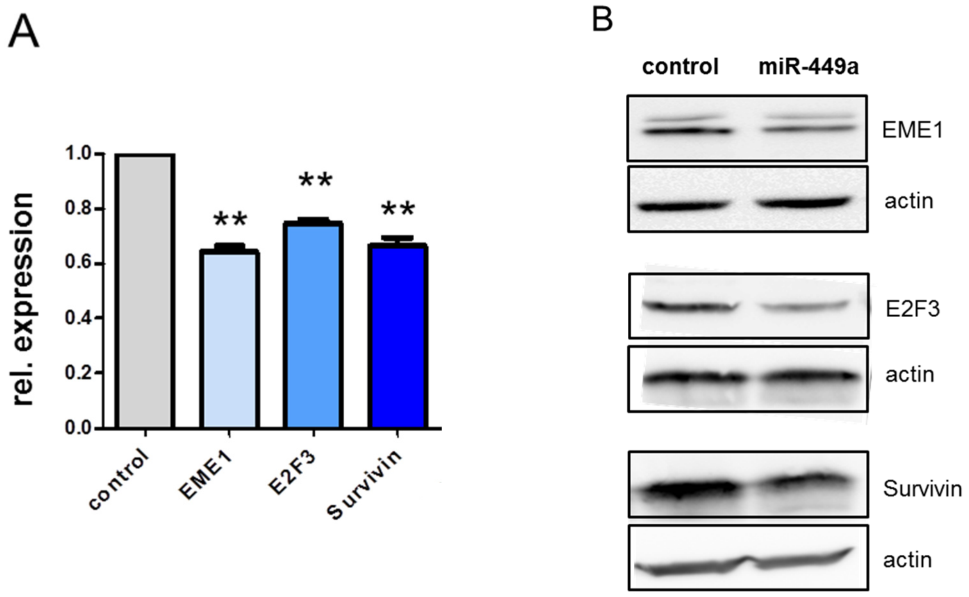

Figure S5: Validation of miR449a-mediated knockdown of EME1, E2F3 and Survivin in TNBC cell line HCC1937 (**A**) Expression of *EME1*, *E2F3*, and *Survivin* mRNA measured by RT-PCR 48 h after transfection with miR-449a mimics or control. (**B**) Expression of EME1, E2F3 and Survivin protein using Western blotting in HCC38 cells. Error bars represent SEM. n=3. **P < 0.01, 2-tailed Student's t test.

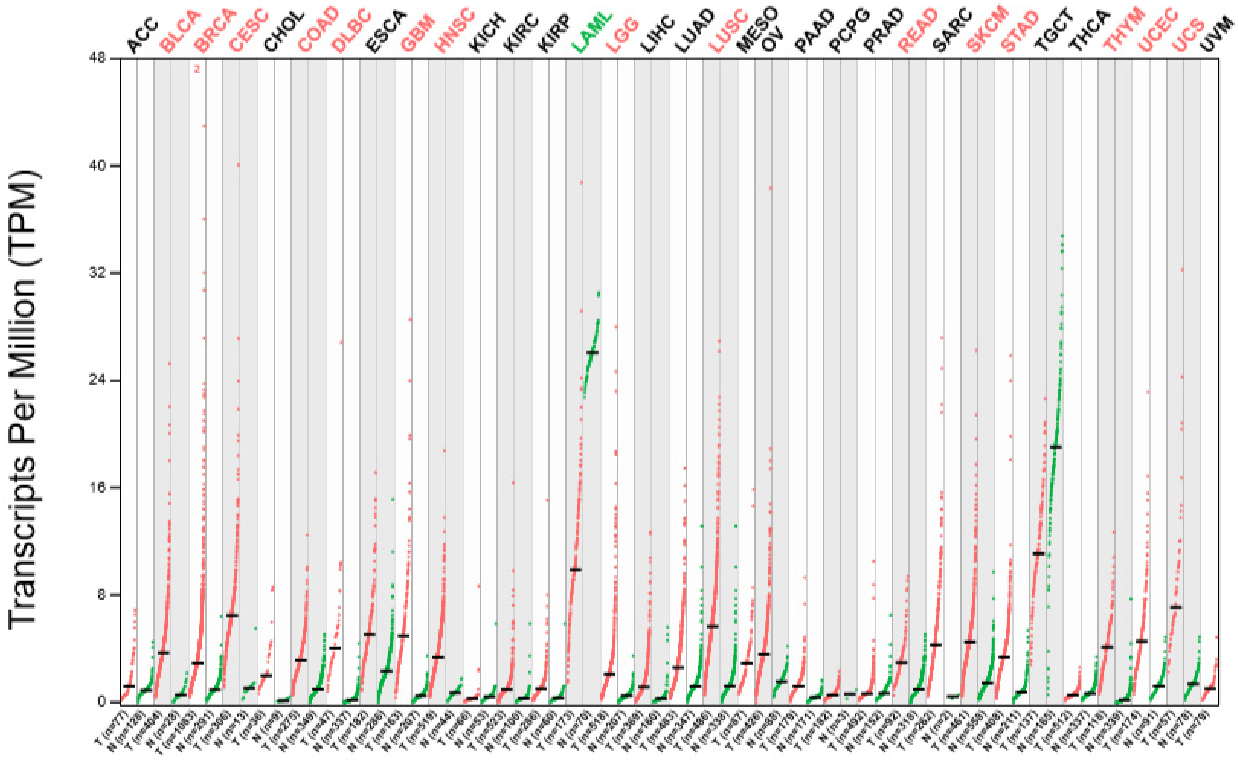

Figure S6: *EME1* transcripts in different tumor samples vs normal samples derived from The Cancer Genome Atlas

TCGA data analyses were performed with the Gene Expression Profiling Interactive Analysis (GEPIA) (18). The gene expression profile is shown across all tumor samples and paired normal tissues. Each dot represent *EME1* expression per samples. T: tumor, N: normal. In red: cohorts with higher expression of *EME1* in tumor compared to normal samples as BLCA (Bladder Urothelial Carcinoma), BRCA (Breast invasive carcinoma), CESC (Cervival squamous cell carcinoma and endocervical adenocarcinoma), COAD (Colon carcinoma), DLBC (Lymphoid neoplasm diffuse large B-cell lymphoma), GBM (Glioblastoma multiforme), HNSC (Head and Neck squamous cell carcinoma), LGG (Brain lower grade glioma), LUSC (Lung squamous cell carcinoma), READ (Rectum adenocarcinoma), SKCM (Skin cutaneous melanoma), STAD (stomach adenocarcinoma), THYM (Thymoma), UCEC (Uterine corpus endometrial carcinoma) and UCS (Uterine carcinosarcoma). In green: LAML (Acute meyloid leukemia) cohort with higher expression of EME1 in normal compared to tumor samples.

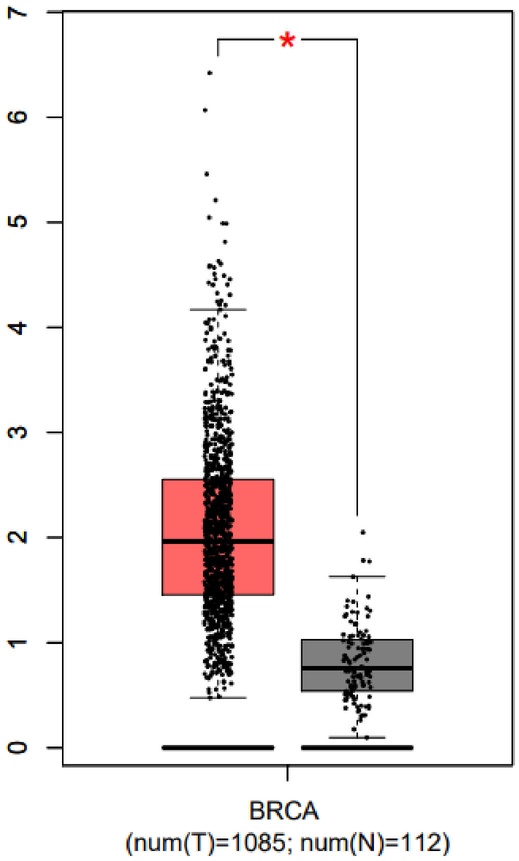

Figure S7: *EME1* expression in tumor samples vs normal samples derived from The Cancer Genome Atlas. TCGA data analyses were performed with web server GEPIA (18). The gene expression profile is shown for tumor samples and paired normal tissues of the BRCA (breast invasive carcinoma) cohort. Each dot represent expression in a single sample. T: tumor, N: normal. *P < 0.01

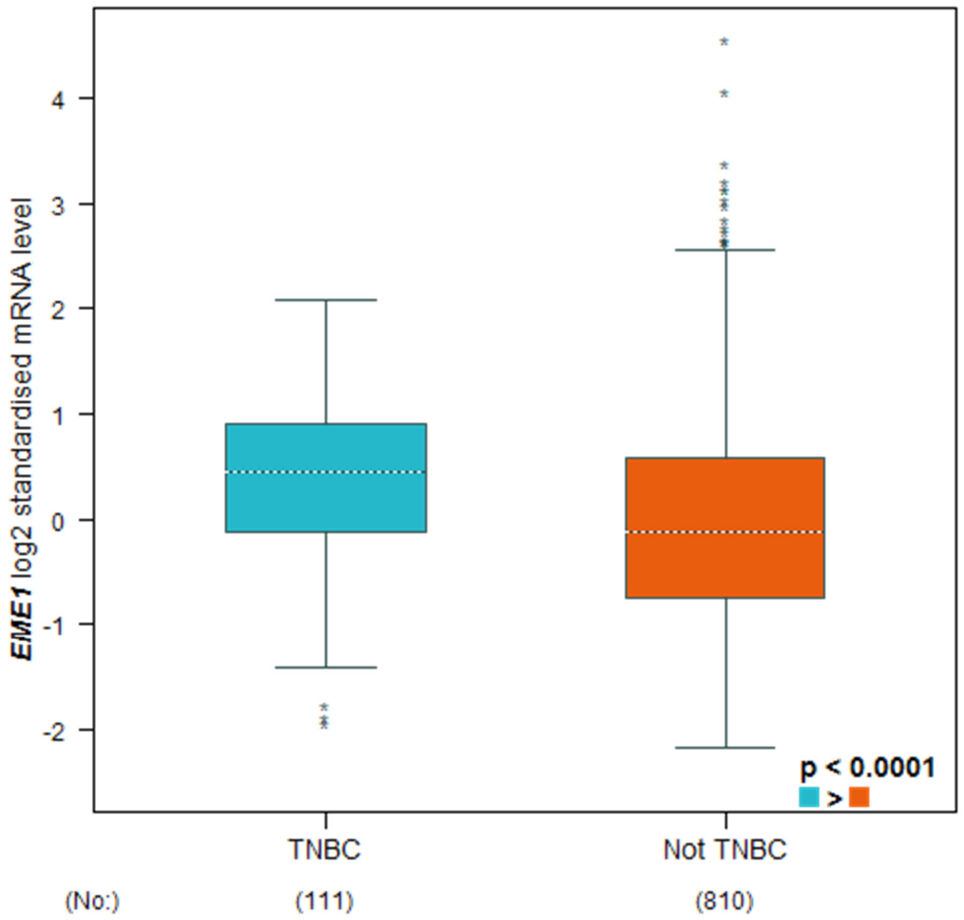

Figure S8: *EME1* expression in TNBC samples vs Not TNBC samples derived from The Cancer Genome Atlas

TCGA Data analyses were performed with Breast Cancer Gene-Expression Miner v4.3 (13). Box and whisker plot of EME1 expression, asterisks show outlier. N= 921 patients. P < 0.0001

A

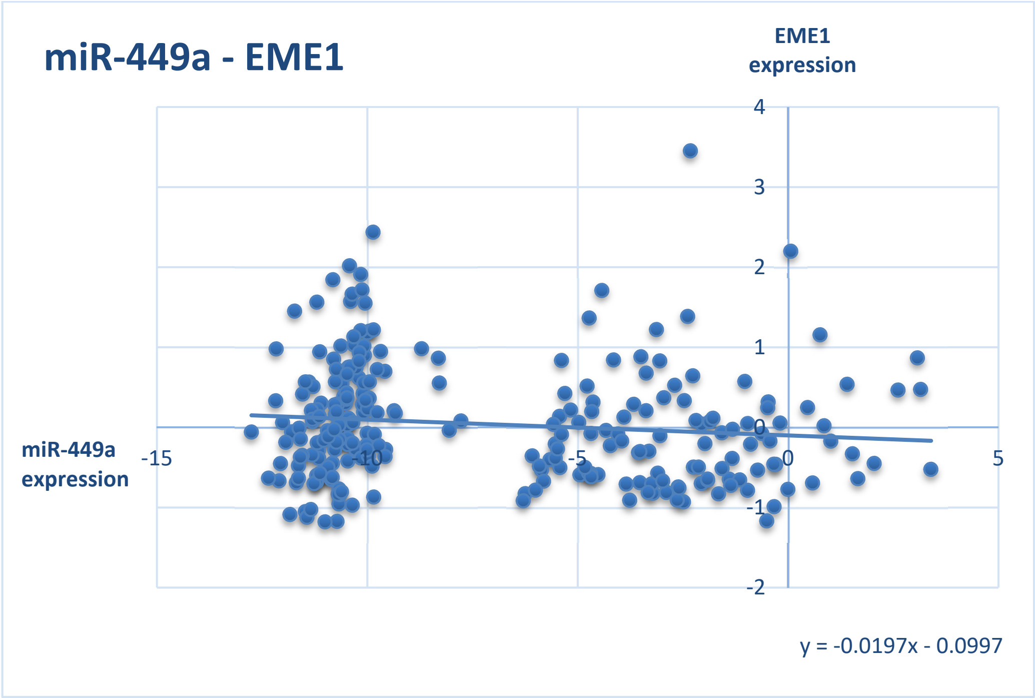

B

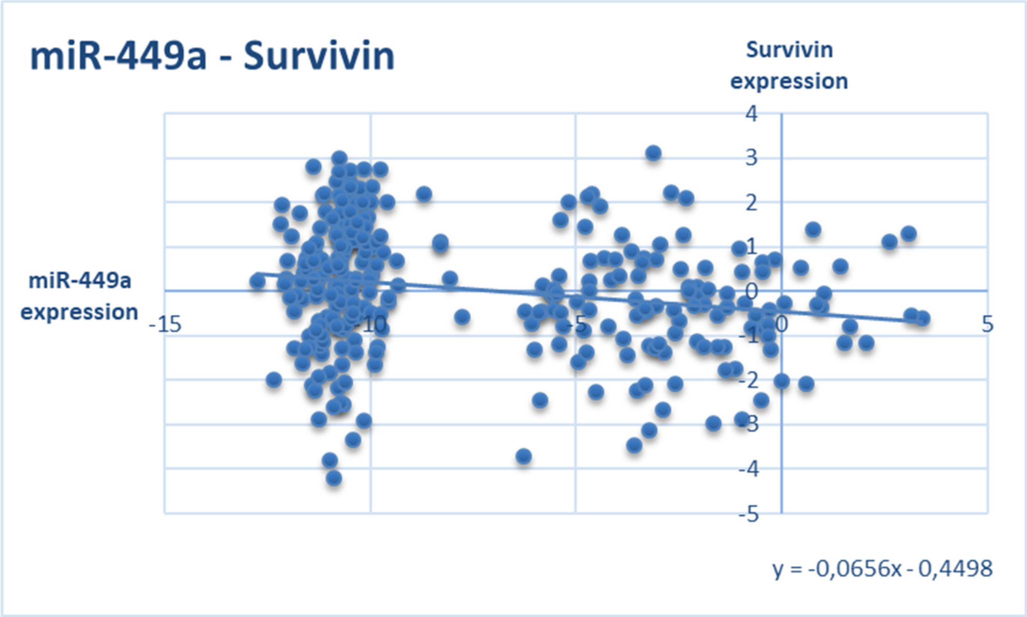

Figure S9: Correlation of gene expression of miR-449a and EME1 or Survivin. (**A**) Correlation of gene expression of miR-449a and EME1 in the dataset GSE58215. Gene expression was analyzed with GEO2R of the NCBI. The dataset GSE58215 provided expression of miRNAs as well as gene expression in breast cancer (n=258). (**B**) Correlation of gene expression of miR-449a and Survivin in the dataset GSE8215. The correlation is calculated using a linear regression.

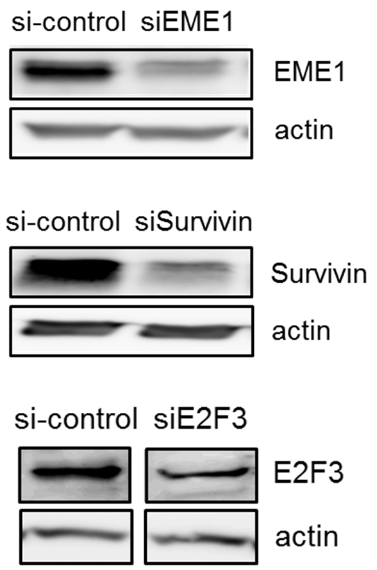

Figure S10: Validation of si-mediated knockdown of EME1, Survivin and E2F3 on protein level

Expression of EME1, Survivin and E2F3 protein in HCC38 cells after transfection of si-RNAs using Western blotting. Actin was used as a loading control. Si-Pools were used. n=3

Supplementary Methods

Cell culture

The triple-negative breast cancer cell lines HCC38, HCC1937 and HCC1395 (ATCC) were cultivated in T25, T75 or T175 cell culture flasks (Sarstedt) at 37°C and 5% CO_2 in a humidified incubator. All three cell lines were supplied with RPMI-1640 growth medium (Sigma-Aldrich) with 10 % fetal bovine serum (Biochrom), 1 % penicillin/streptomycin (Biochrom), 12,5 mL 10 % D-glucose solution (Sigma-Aldrich), 1 % HEPES and 1 % sodium pyruvate (PAA / GE Healthcare).

For transfections, HiPerFect Transfection Reagent (Qiagen) was used according to the manufacturer's instructions. All cell lines were seeded and transfected simultaneously. The medium was changed 24 h after transfection.

Table S3: SiRNAs and miRNA mimics for transfection.

**Oligonucleotide**	**Manufacturer**

Allstars Negative Control SI03650318	Qiagen, Hilden, Germany
syn-hsa-miR-449a MSY0001541	Qiagen, Hilden, Germany
syn-hsa-miR-449b-5p MSY0003327	Qiagen, Hilden, Germany
syn-hsa-miR-449c-5p MSY0010251	Qiagen, Hilden, Germany
Negative Control siPOOL N000-034	siTools Biotech, Planegg, Germany
siPOOL BIRC5	siTools Biotech, Planegg, Germany
siPOOL EME1	siTools Biotech, Planegg, Germany
siPOOL E2F3	siTools Biotech, Planegg, Germany

Chromatin immunoprecipitation

After TSA treatment of TNBC cells for 1 h, DNA and proteins were crosslinked with 1% formaldehyde for 15 min by adding 540 µL formaldehyde (37%) to 20 mL cell culture medium. The reaction was stopped by adding 1 mL 2.5 M glycine (final concentration of 0.125M). Cells were washed twice with 10 mL PBS. After adding another 10 mL PBS, cells were scraped from the cell culture dish, transferred to a 50 mL tube and centrifuged at 4000 rpm for 1 min. The supernatant was removed and the cell pellet was lysed in 500 µL lysis buffer. The lysates were sonicated (Covaris S2/E210, Covaris, Woburn, MA, USA) with following settings: 5% duty cycle, intensity 3, 200 cycles/burst, 80 sec. The fragment size of sheared DNA was checked on an agarose gel (fragment size around 500 bp). The sonicated lysates were centrifuged at maximum speed for 10 min at 4 °C to remove cell debris. The supernatant containing the sonicated DNA was diluted 1:6 with dilution buffer and precleared overnight with 40 µL protein G magnetic beads (Merck, Darmstadt, Germany). The next day, the beads were removed. 75 µg DNA and 3 µL antibody per reaction were combined and rotated for 6 h at 4 °C. Then, 40 µL beads per reaction were added and incubated at 4°C overnight. After washing the beads with 1 mL TSE I, TSE II and buffer III, protein/DNA-complexes were eluted twice with 100 µL elution buffer. Crosslinking was reversed by incubation at 65°C overnight. DNA was purified with a PCR purification kit (Qiagen) and quantitative PCR was performed using 2 µL sample and primers specific for the CDC20B promoter.

<u>lysis buffer</u>
1% SDS
10 mM EDTA
50 mM Tris-HCl pH7.8

<u>dilution buffer</u>
1% Triton
2 mM EDTA
150 mM NaCl
20 mMTris-HCl pH7.8

<u>TSE I</u>
150 mM NaCl
0.1% SDS

1% Triton
2 mM EDTA
20 mM Tris-HCl pH7.8

<u>TSE II</u>
500 mM NaCl
0.1% SDS
1% Triton
2 mM EDTA
20 mM Tris-HCl pH7.8

<u>buffer III</u>
0.25 M LiCl
1% NP-40
1% deoxycholate
1 mM EDTA
10 mM Tris-HCl ph7.8

<u>elution buffer</u>
0.1 M $NaHCO_3$
1% SDS

Table S4: Antibodies for chromatin immunoprecipitation.

**Antigen**	**Order no.**	**Manufacturer**
H3	ab1791	Abcam, Cambridge, UK
H3K9ac	9649S	Merck Millipore, Darmstadt, Germany
H4	ab10158	Abcam, Cambridge, UK
H4K5ac	07-327	Merck Millipore, Darmstadt, Germany
H4K8ac	07-328	Merck Millipore, Darmstadt, Germany
H4K12ac	07-595	Merck Millipore, Darmstadt, Germany
H4K16ac	07-329	Merck Millipore, Darmstadt, Germany
Rabbit IgG	2729S	Cell Signaling, Leiden, Netherlands

PTEMF buffer
20 mM PIPES pH 6.8
10 mM EGTA
0.2% Triton X-100
1 mM $MgCl_2$
4% formaldehyde

Table S5: Primers for quantitative real-time PCR after chromatin immunoprecipitation.

Primer	Sequence
CDC20B-1 fw	5'-TAGCTGACACAGCGATTACCAACG-3'
CDC20B-1 rv	5'-TTCTCTTTCTCCAACCACACCCTG-3'

RNA immunoprecipitation

MiR-449a versus control transfected HCC38 cells were lysed in 3x NP40 lysis buffer. The cells were incubated for 20 min on ice. Then, the cells were pelleted by centrifugation (15 min, 13.000 g and 4°C). For input 20 µl of the supernatant was stored. Dynabeads (Invitrogen) were prepared by 3x washing with citrate-phosphate buffer and 1 h incubation at 4°C with control IgG or Ago2 antibody. The cell lysates were incubated with dynabeads for 1 h at 4°C. To control and monitor successful immunoprecipitation (IP), an aliquot of beads was taken and Ago2 pulldown was analysed by Western Blot. After successful IP, RNA was isolated by the use of TRIZOL (Sigma-Aldrich). Isolated RNA from Ago2-IP was subjected to qPCR for validation.

Table S6: Antibodies for RNA immunoprecipitation

Antigen	Order no.	Manufacturer
Argonaute 2 rat mAB	11A9	Chromotek, Planegg-Martinsried, Germany
IgG2A rat	3E8	Chromotek, Planegg-Martinsried, Germany

1x NP40 lysis buffer

50 mM Hepes-KOH pH 7,5

150 mM KCl

2 mM EDTA

1mM NaF

0.5 % Igepal

0.5 mM DTT

+ 1x 25x Complete

Citrat-phosphate buffer pH 5,0

25 mM citric acid

65 mM Na_2HPO_4

IP-wash buffer, pH 7,5

50 mM Hepes-KOH pH 7,5

300 mM KCl

0,05 % Igepal

0.5 mM DTT

+ 1x 25x Complete

Global mRNA expression profiling

Global mRNA expression profiling was performed as previously described (Sandbothe) utilizing microarrays and the SurePrint G3 Human Gene Expression v2 8x60K Microarray Kit (Agilent, Santa Clara, CA, USA) according to manufacturer's instructions. In brief, RNA of four biological replicates was labeled using the Low Input Quick Amp Labeling Kit (Agilent). After hybridization, washing, and scanning of the arrays signal intensities were extracted with Feature Extraction software (Agilent) and normalized by percentile shift to 75th percentile. Differentially expressed genes were identified by the following criteria: fold change ≥ 1.5 and $P < 0.25$ as determined by unpaired t-test with Benjamini-Hochberg correction. Pathway analyses of differentially expressed genes were performed with a cutoff of $P < 0.05$. All microarray analyses were performed with the GeneSpring GX software (Agilent).

Expression analysis of mRNA and miRNA

To isolate RNA, cells were lysed in Qiazol Lysis Reagent (Qiagen) and total RNA including miRNAs was isolated with the miRNeasy Mini Kit (Qiagen). RNA quality was routinely determined using the Bioanalyzer 2100 (Agilent, Santa Clara, CA, USA). Expression of mRNA and miRNA was measured in triplicate by quantitative real-time PCR (qRT-PCR). Complementary DNA (cDNA) was reverse transcribed with the High Capacity cDNA Transcription Kit (Life Technologies). For mRNA, cDNA synthesis was performed with 250 ng total RNA using random hexamer primers. Taqman Gene Expression Assays (Life Technologies) were used in qRT-PCR with *TBP* as reference gene. For miRNA, Taqman MicroRNA Assays (Life Technologies) were used for synthesis of miRNA-specific cDNA from 100 ng total RNA and qRT-PCR was performed with *RNU6B* as reference gene.

Table S7: Taqman Assays for quantitative real-time PCR.

**Target gene / miRNA**	**Assay ID**	**Manufacturer**
E2F3	Hs00605457_m1	Thermo Fisher Scientific, Braunschweig, Germany
BIRC5	Hs04194392_s1	Thermo Fisher Scientific, Braunschweig, Germany
TBP	Hs00920494_m1	Thermo Fisher Scientific, Braunschweig, Germany
BRCA2	Hs00609073_m1	Thermo Fisher Scientific, Braunschweig, Germany
RAD51	Hs00947967_m1	Thermo Fisher Scientific, Braunschweig, Germany
EME1	Hs01103357_g1	Thermo Fisher Scientific, Braunschweig, Germany
miR-449a	1030	Thermo Fisher Scientific, Braunschweig, Germany
miR-449b	1026	Thermo Fisher Scientific, Braunschweig, Germany
miR-449c	241086_mat	Thermo Fisher Scientific, Braunschweig, Germany
RNU6B	1093	Thermo Fisher Scientific, Braunschweig, Germany

Expression analysis of protein

Table S8: Antibodies for western blotting.

**Primary antibody**	**Manufacturer**	**Dilution**
Anti beta-actin 3700	Cell Signaling Technology, Leiden, NL	1:2000
Survivin (2808)	Cell Signaling Technology, Leiden, NL	1:1000
E2F3 (pa2142)	Boster Bio Pleasanton, CA , USA	1:1000
Eme1 (MTA31 7h2/1): sc-53275	Santa Cruz Biotechnology (Dallas, USA)	1:200
Rad51 (D4B10) Rabbit mAb	Cell Signaling Technology, Leiden, NL	1:1000
Anti-BRCA2 antibody (ab123491)	Abcam (Cambridge, UK)	1:10000

Secondary antibody		
Goat Anti-Mouse IgG (HRP) (554002)	BD Biosciences (San Jose, CA, USA)	
Goat Anti-Rabbit IgG (HRP) **(**ab6721)	Abcam (Cambridge, UK)	

Table S9: Antibodies for immunofluorescence.

**Primary antibody**	**Manufacturer**	**Dilution**
Anti-phospho-Histone H2A.X (Ser139) Antibody, clone JBW301	Merck (Darmstadt, DE)	1:1000
Anti-PICH **(**clone 142-26-3)	Merck (Darmstadt, DE)	1:50
53BP1 (6B3E10): sc-517281	Santa Cruz Bio-technology (Dallas, USA)	1:200
**Secondary antibody**		
Alexa Fluor^{TM} 488 goat anti-mouse IgG	Thermo Fisher Scientific (Waltham, USA)	

Cloning of Luciferase Reporter Vectors

Table S10: Primers for cloning of luciferase reporter vectors.
(Overhang sequences to generate *Xba*I restriction sites are indicated in italics. Predicted binding sites of the miR-449 family are underlined, mutated bases are indicated in bold.)

**Primer**	**Sequence**
EME1-3’UTR-WT (gBlock)	GAT ATA TCT AGA TTC TAG CCC TCA GGG ATG AGG ATG AAA AGC TGG AAA CTT CCA CTT CCC CAA CCT CAG AGC CTG ACT GTA ATG AAG AGA CTG GCA GCA CCT CCT GGA ACA CAA GCC TAG GTG AGG CCC AGT CTT TCT TGG GTC TTA TTA TTT GTG AAG GTC TCT CTG CCT GTC GGC TGG GGC AGA GAC TGA AAT ACT GCC ACC TAC CTT TGG CAT TTA ATG TTC CTC TCC TGG CAA AAA TTC ACT GCC ACA GAC AAA CCA CCC CCA CTC CTA CCC AGC CAG CCC TCA AAA CAC AAA GGA ACA AAG ACA GTC CAC TCA GAC ACT TAT TTA ATA ACT GTA GAA ATC CAA AAG AAT TAG CAT CAA ATC TTG AAG TCG TGA GTG AAG CTG CGG
EME1-3’UTR-mut (gBlock)	GAT ATA TCT AGA TTC TAG CCC TCA GGG ATG AGG ATG AAA AGC TGG AAA CTT CCA CTT CCC CAA CCT CAG AGC CTG ACT GTA ATG AAG AGA CTG GCA GCA CCT CCT GGA ACA CAA GCC TAG GTG AGG CCC AGT CTT TCT TGG GTC TTA TTA TTT GTG AAG GTC TCT CTG CCT GTC GGC TGG GGC AGA GAC TGA AAT GCA GTC GCC TAC CTT TGG CAT TTA ATG TTC CTC TCC TGG CAA AAA TTC GCA GTC GCA GAC AAA CCA CCC CCA CTC CTA CCC AGC CAG CCC TCA AAA CAC AAA GGA ACA AAG ACA GTC CAC TCA GAC ACT TAT TTA ATA ACT GTA GAA ATC CAA AAG AAT TAG CAT CAA ATC TTG AAG TCG TGA GTG AAG CTG CGG
